# Fluorescence-based thermometry for precise estimation of nanoparticle laser-induced heating in cancerous cells at nanoscale

**DOI:** 10.1515/nanoph-2022-0314

**Published:** 2022-08-15

**Authors:** Oleksii O. Peltek, Eduard I. Ageev, Pavel M. Talianov, Anna D. Mikushina, Olga S. Epifanovskaya, Aliaksei Dubavik, Vadim P. Veiko, Kirill Lepik, Dmitry A. Zuev, Alexander S. Timin, Mikhail V. Zyuzin

**Affiliations:** School of Physics and Engineering, ITMO University, Lomonosova 9, 191002, St. Petersburg, Russian Federation; Laboratory of Renewable Energy Sources, Alferov University, Khlopina 8/3, 194021, St. Petersburg, Russian Federation; RM Gorbacheva Research Institute of Pediatric Oncology, Hematology and Transplantation, Pavlov University, Lva Tolstogo 6/8, 191144, St. Petersburg, Russian Federation; Faculty of Photonics, Center of Optical Information Technologies, ITMO University, Birzhevaya liniya 4, 199034, St. Petersburg, Russian Federation

**Keywords:** fluorescent nanothermometry, laser-induced heating, necrosis/apoptosis, photodynamic therapy

## Abstract

Photothermal therapy (PTT) has attracted increasing interest as a complementary method to be used alongside conventional therapies. Despite a great number of studies in this field, only a few have explored how temperatures affect the outcome of the PTT at nanoscale. In this work, we study the necrosis/apoptosis process of cancerous cells that occurs during PTT, using a combination of local laser heating and nanoscale fluorescence thermometry techniques. The temperature distribution within a whole cell was evaluated using fluorescence lifetime imaging microscopy during laser-induced hyperthermia. For this, gold nanorods were utilized as nanoheaters. The local near-infrared laser illumination produces a temperature gradient across the cells, which is precisely measured by nanoscale thermometry. This allows one to optimize the PTT conditions by varying concentration of gold nanorods associated with cells and laser power density. During the PTT procedure, such an approach enables an accurate determination of the percentages of apoptotic and necrotic cells using 2D and 3D models. According to the performed cell experiments, the influence of temperature increase during the PTT on cell death mechanisms has been verified and determined. Our investigations can improve the understanding of the PTT mechanisms and increase its therapeutic efficiency while avoiding any side effects.

## 1 Introduction

Malignant skin neoplasms, especially melanomas, are highly aggressive and result in approximately 65% of all skin cancer deaths and a long-term survival rate of 5% [1]. At the moment, the main therapeutic approaches used for the treatment of melanoma include chemotherapy, surgery, immunotherapy, and radiotherapy [2–7]. However, these methods of treatment are not always satisfying due to possible side effects, cell-resistance to the chemotherapeutic drugs, and radioactive emission [8–11].

As an alternative, photothermal therapy (PTT) can be considered as an extensively developing method of cancer treatment [12–15]. This technique requires the use of light-sensitive nanoscale objects (e.g., biomolecules, nanoparticles etc.) that can convert light energy into heat. By applying the laser irradiation to the zone of interest (e.g., the tumor with impregnated light-responsive agents), it induces hyperthermia, which leads to the death of cancerous cells [16–18]. The main advantages of the PTT include spatiotemporal selectivity of treatment, enhanced immunogenicity, and lack of the protective mechanisms of cancer cells [19–21].

At present, the number of preclinical and clinical trials on the use of PTT for melanoma treatment is rapidly growing. There are already several completed clinical trials on PTT. For example, NCT00848042 for refractory and/or recurrent head and neck tumors (2008–2014), NCT01679470 for metastatic lung tumors (2012–2014), and NCT02680535 for localized prostate cancer (2016–2021). All these clinical trials employed AuroLase Au NPs as a photosensitive agent for systemic administration [22]. The latest clinical trial on the use of PTT has recently demonstrated results for the treatment of localized prostate cancer (NCT02680535). As a result of the PTT, 15 out of 16 patients fully recovered, and a year later, a biopsy confirmed no relapse. These data speak in favor of the possibility of using PTT not only for the treatment of malignant tumors near the skin surface, but also for the treatment of malignancies in the deeper tissues, using optical fiber for supplying radiation.

In order to improve the efficiency of PTT cancer treatment, an understanding of different cell death mechanisms is crucial. The cell death induced by hyperthermia usually occurs via two possible pathways: necrosis or apoptosis [23–25]. Apoptosis is referred to as programmed cell death. In contrast, necrosis is an uncontrolled form of cell death that is induced by external injuries and in most cases is caused by membrane rupture. During cancer therapy, the failure to regulate the apoptosis/necrosis process can result in severe adverse effects [26]. During PTT, the cell death pathway depends on the intracellular temperature induced by laser heating [27, 28]. Thus, it is important to accurately measure and control the intracellular temperature reached during the PTT treatment.

There are a number of works devoted to cell thermometry at the nanoscale level. The main approaches of nanothermometry mostly utilize organic dyes, quantum dots, upconverting nanoparticles, and nanodiamonds [29–33].

However, despite the great number of available methods, to the best of our knowledge, there are no works that managed to measure the intracellular temperature distribution during the PTT. The closest works on this subject describe the effect of PTT based on the temperature of the cell media that contained gold nanorods (Au NRs), during laser irradiation using infrared thermometry [23, 34]. However, we believe that different approach can be used for intracellular temperature distribution measurements to obtain temperatures directly within a cell.

**Figure 1:**
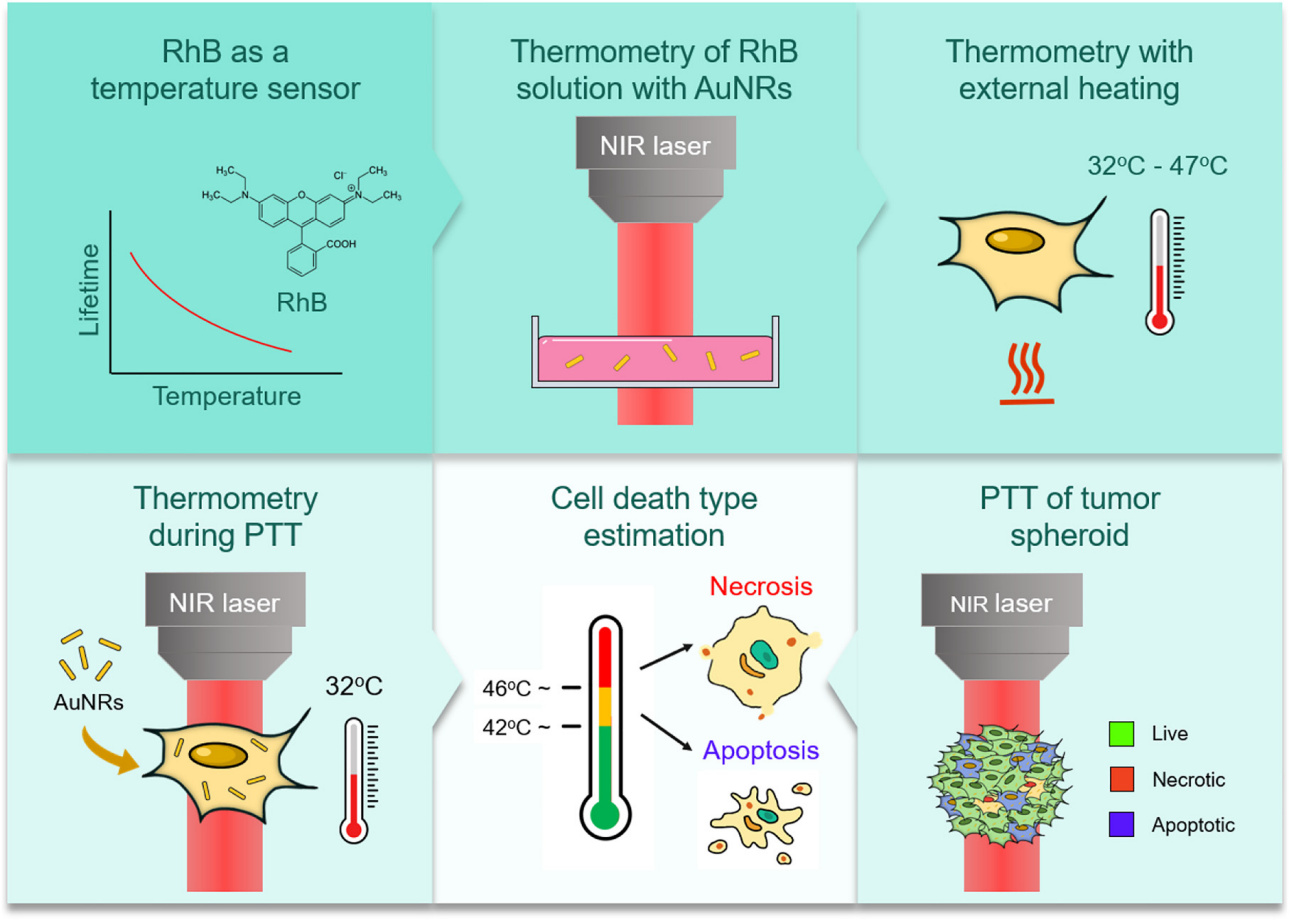
Schematic illustration of roadmap in this work: (i) estimation of temperature dependence of RhB fluorescence lifetime versus the applied temperature; (ii) temperature estimation of laser-induced heating of RhB; (iii) thermometry of an individual cell with applied external heating; (iv) measuring of temperatures of 2D cell model under laser-induced heating; (v) evaluation of the percentage ratio of necrotic and apoptotic cells under laser-induced heating; (vi) evaluation of the percentage ratio of necrotic and apoptotic cells in tumor spheroid under laser-induced heating.

To achieve this goal, we decided to apply organic dye (Rhodamine B, RhB) for fluorescent lifetime-based thermometry due to the simplicity of temperature measurements and easy access to the organic dyes in almost every chemical laboratory. Au NRs were used as nanoheaters when performing PTT using a near-infrared laser (NIR, 1064 nm fiber laser, 100 kHz). To evaluate the cell death pattern apoptosis and necrosis assay using flow cytometry was performed. Additionally, the change in Bax gene expression was investigated in irradiated cells, as it encodes apoptosis-inducing protein BCL2L4 and its expression was elevated in cells subjected to heat-induced apoptosis [35–37]. The roadmap of the described study is presented in Figure 1.

We believe the results of this work will enable a better understanding of the influence of the intracellular temperatures reached during the PTT on cell death pattern. Additionally, the reported findings can serve to increase the efficiency of PTT and decrease the adverse effects that may occur during this procedure. Furthermore, we believe that the use of methods for intracellular temperature estimation is not limited only to PTT, but they can find their application in other fields of knowledge.

## 2 Experimental section

### 2.1 Materials

*For Au NRs synthesis*: gold (III) chloride trihydrate (HAuCl_4_·3H_2_O, ≥99.9%, Sigma-Aldrich), ascorbic acid (AA, ≥99.0%, Sigma-Aldrich), sodium borohydride (NaBH_4_, 98%, Sigma-Aldrich), cetyltrimethylammonium bromide (CTAB, ≥99%, Sigma-Aldrich), sodium oleate (NaOL, > 97.0%, Sigma-Aldrich), and amine-poly(ethylene glycol)-thiol (NH_2_-PEG-SH, MW 1.000, Laysan Bio, China) were all used without additional purification.

*For cell cultures*: Alpha Minimum Essential Medium (Alpha-MEM) was purchased from Biolot, Russia. Phosphate-buffered saline (PBS), and UltraGlutamine I were purchased from Lonza, Switzerland. Fetal bovine serum (FBS) was obtained from HyClone, USA. Trypsin-EDTA solution was purchased from Capricorn Scientific, Germany. Rhodamine B (RhB, ≥95%) and calcein acetoxymethyl (Calcein AM) were purchased from Sigma-Aldrich. AlamarBlue cell viability reagent was purchased from Invitrogen, USA. APC Annexin V apoptosis detection kit with 7-AAD was purchased from BioLegend, USA.

### 2.2 Synthesis of functionalized Au NRs

Au NRs were synthesized using seed-mediated growth approach and binary surfactant mixture according to the protocol developed by Xe et al. [38] Surface coating of CTAB-stabilized Au NRs was realized with ligand exchange procedure [39]. Details and the full data set are given in the Supporting Information.

### 2.3 Characterization of Au NPs

The absorption spectra for the aqueous solutions of the functionalized Au NRs were measured in the 10 mm path quartz cuvettes using spectrophotometer Shimadzu UV-3600 (400–1300 nm interval). Size distribution of the Au NPs was estimated using the scanning electron microscopy (SEM, Carl Zeiss Merlin) at an accelerating voltage of 30 kV. The sample preparation procedure and technical details are presented in the Supporting Information.

### 2.4 Experimental setup for fluorescence lifetime measurements

To measure the fluorescence lifetimes, time-correlated single photon counting (TCSPC) method was used. The experimental setup is based on a home-built confocal microscope for fluorescence lifetime measurements with TCSPC. To provide the heating of gold nanorods, an additional ytterbium laser source (1064 nm fiber laser, 100  kHz, <140 ns FWHM) was used. The detailed data and optical scheme are described in the Supporting Information.

### 2.5 Cells

Murine melanoma cell line (B16-F10 cells) was obtained from the American Type Culture Collection. Cells were cultured in AlphaMEM supplemented with 10% of vol. FBS and additional 2 mM UltraGlutamine I. The cell culture was maintained in a sterile humidified atmosphere containing 95% air and 5% CO_2_ at 37 °C.

### 2.6 Toxicity studies

In order to evaluate the toxicity of Au NRs at different concentrations, AlamarBlue assay was performed. The cell viability was analyzed by measuring absorbances of the media at 570 and 600 nm with UV−vis spectrophotometer (Thermo Scientific Multiskan GO). The detailed protocols are presented in the Supporting Information.

### 2.7 Au NRs uptake

To evaluate uptake and association of Au NRs with B16-F10, cells were incubated with Cy-5 labelled Au NRs. Afterwards uptake was visualized using confocal laser scanning microscopy. The data and detailed experiment description are presented in the Supporting Information.

### 2.8 External heating of Rhodamine B (RhB) solution

In order to obtain a calibration curve describing the dependence of fluorescence lifetime of RhB on the temperature, 100 µM of aqueous RhB solution was used. Values of fluorescence lifetime were obtained using Picoquant Picoharp 300 time-correlated single photon counting system. Heating of RhB solution was realized using a custom made temperature control system, where the temperature of the solution was monitored via a thermocouple. The values of the fluorescence lifetimes of RhB were measured at various temperatures. The obtained data were fitted by single exponential fit using Matlab software. This fit was later used as a calibration curve to estimate the RhB solution temperature based on the measured fluorescence lifetime values. The details and the full data set are given in the Supporting Information.

### 2.9 Laser-induced heating of Au NRs in RhB solution

To evaluate the laser-induced heating of Au NRs, a freshly prepared 100 μM solution of RhB containing Au NRs with a final concentration of 20 μg/mL was used. Fluorescence lifetime measurements were carried out on the custom-made setup. In order to evaluate the laser-induced heating of Au NRs in RhB solution, it was irradiated with a pulsed 1064 nm laser with different power densities (up to 36.3 kW/cm^2^). Before each measurement, the solution was continuously irradiated for 60 s to stabilize the temperature. In order to estimate the temperatures of the solution during laser treatment, we used the previously obtained calibration curve. Using this dependence, we recalculated the measured fluorescence lifetimes into their corresponding temperature values. The results and calculations are given in the Supporting Information.

### 2.10 External heating of cells

To estimate local cell temperatures during external heating, cells were seeded on confocal cell imaging dishes. Next day, Au NRs were added to the cells, and the cells were left overnight. On the following day, the cells were stained with RhB. For this, 10 µL of 1 mM RhB solution was added to the cell culture medium, so that the final concentration of RhB was equal to 10 µM. After 30 min, cells were washed twice with PBS to remove non-internalized Au NRs and left in PBS supplemented with 4% glucose. Fluorescence lifetime measurements were performed with custom-made setup. Before measurements, cells were heated up to a certain temperature (32–47 °C) with a custom-made temperature control system to ensure the uniformity of temperature distribution in the cell imaging dish. The obtained data was fitted by single exponential fit using Matlab software. This fit was later used as a calibration curve to estimate mean intracellular temperature based on the measured fluorescence lifetime values. The detailed protocols and obtained data are presented in the Supporting Information.

### 2.11 Laser-induced heating of gold nanorods in cells

In order to heat up cells with an NIR-laser, the cells were seeded into confocal cell imaging dishes with Au NRs at three different final concentrations of Au NRs in the culture media (20, 40 and 60 μg/mL). On the following day, the cells were stained with RhB. After 30 min, the cells were washed twice with PBS and left in PBS supplemented with 4% glucose. Fluorescence lifetime measurements were performed with the setup described in Section 2.10. During these measurements, the temperature of the cell medium was kept at 32 °C. Prior to the measurement, the cells were continuously irradiated for 60 s to stabilize the temperature. The previously obtained fit was used in these measurements as a calibration curve to estimate mean intracellular temperature based on the measured fluorescence lifetime values.

### 2.12 Flow cytometry

The apoptosis/necrosis assay was performed using flow cytometry (FACS Aria, BD, USA). For this, cells were seeded in 12-well plates at the amount of 1.0 × 10^5^ per well. Next day, Au NRs were added to each well at different concentrations (20 μg/mL to 60 μg/mL). The final volume of cell culture medium in each well was 1 mL. Afterwards, cells were washed twice with PBS and left in PBS supplemented with 4% glucose. Then, each well was irradiated with an NIR-laser with different power densities (up to 43.2 kW/cm^2^) for 90 s. One hour after the irradiation, the cells were detached using trypsin-EDTA (*V* = 200 µL per well) solution and stained with 7-AAD and APC Annexin V according to the protocol provided by the manufacturer (BioLegend, USA).

### 2.13 Bax gene expression analysis

The effect of induced hyperthermia on cell apoptosis was further evaluated by estimating the increase of Bax gene expression using real-time polymerase chain reaction (PCR). For this, cells were seeded in 12-well plates at the amount of 1.0 × 10^5^ cells per well. Next day, Au NRs were added to each well at different concentrations (20 μg/mL to 60 μg/mL) and incubated overnight at 37 °С, 5% CO_2_. Afterwards, cells were washed twice with PBS and left in PBS supplemented with 4% glucose. Then, each well was irradiated with an NIR-laser with different power densities (up to 43.2 kW/cm^2^) for 90 s. One hour after the irradiation, total RNA was extracted and purified using an RNA extraction kit (Evrogen, Russia). Afterwards cDNA was synthesized from total RNA and real-time PCR was carried out. The detailed protocols are described in the Supporting Information.

### 2.14 Formation of tumor spheroid

Spheroids were obtained using the “hanging drop” technique described by Timmins et al. [40, 41] Briefly, 15 μL of culture medium which contained 5000 B16-F10 cell s were carefully dropped onto the inside cover of a 35 mm Petri dish. The dish itself was filled with 1.5 mL PBS to prevent evaporation of cell media. The cover of the dish was placed back on the Petri dish, and the cells were incubated at 37 °C and 5% CO_2_. The cell culture medium inside the drop was changed each 3 days, and after 6 days, the spheroid formation was complete.

### 2.15 Laser-induced heating of Au NRs in spheroid

In order to heat the 3D tumor spheroids with a laser, Au NRs were added to the previously prepared spheroids at a final concentration of 60 μg/mL. The next day, spheroids were washed twice with PBS to remove the non-internalized Au NRs and carefully transferred to a separate Petri dish prior to the irradiation. Then, each spheroid was irradiated for 90 s with an NIR-laser with different power densities (up to 14.2 kW/cm^2^). After the irradiation, each spheroid was stained with calcein AM, APC Annexin V, and 7-AAD. Cells were incubated for 15 min at room temperature before the visualization with a CLSM (Carl Zeiss LSM 710). The detailed protocols and obtained data are presented in the Supporting Information.

## 3 Results and discussion

In this study, we investigate the influence of intracellular temperature induced by PTT on the apoptosis/necrosis ratio of melanoma B16-F10 cells, which was determined by fluorescent lifetime-based thermometry during the PTT procedure. Therefore, we have divided this study into several steps, including (i) estimation of temperature dependence of fluorescence lifetime; (ii) temperature estimation of laser-induced heating; (iii) thermometry analysis of cells at applied external heating; (iv) measuring the temperatures of 2D and 3D cancer cell models under laser-induced heating; and (v) revealing the percentage ratio of necrotic and apoptotic cells during laser-induced heating. The RhB was used for fluorescent lifetime-based thermometry, and Au NRs were applied as nanoheaters for PTT procedure (Figure 1**)**.

### 3.1 RhB as nanothermometer

Thermometry at the nanoscale has gained a lot of interest during recent years as a powerful and versatile tool for biological applications. One of the thermometry approaches is using fluorescent dyes sensitive to changes in the temperature as nanothermometers [42]. This property mainly manifests in molecular rotors (e.g., RhB). The fluorescent properties of RhB depend on the viscosity of the surrounding media, which, in turn, depends on the temperature of the media. In the case of RhB, rotations of the diethylamino groups on the xanthene ring are responsible for the temperature dependence of the fluorescence lifetime and quantum yield [43].

In order to reveal the dependence of fluorescence lifetime on the temperature of the RhB solution, a water solution of RhB (100 μM) was gradually heated from 25 °C to 50 °C with a heating plate, and the fluorescence lifetime values were measured at different temperature points. The fluorescence decay in this and all the further experiments was fitted with a single exponent to determine the values of fluorescence lifetime (Figure S4). The obtained values of fluorescence lifetime are in a good agreement with the previously reported results of 1.74 ns at 20 °C by Boens et al. [44] and the temperature dependence reported by Mercadé-Prieto et al. [45]. The results of our experiments were fitted using exponential fit, which revealed the following dependence:

(1) $$\tau =2.967\cdot {\text{e}}^{-0.0266\cdot T}$$

where *τ* is fluorescence lifetime in nanoseconds (ns) and *T* is temperature in Celsius (°C) (Figure S4).

### 3.2 Au NRs as heating agents

**Figure 2:**
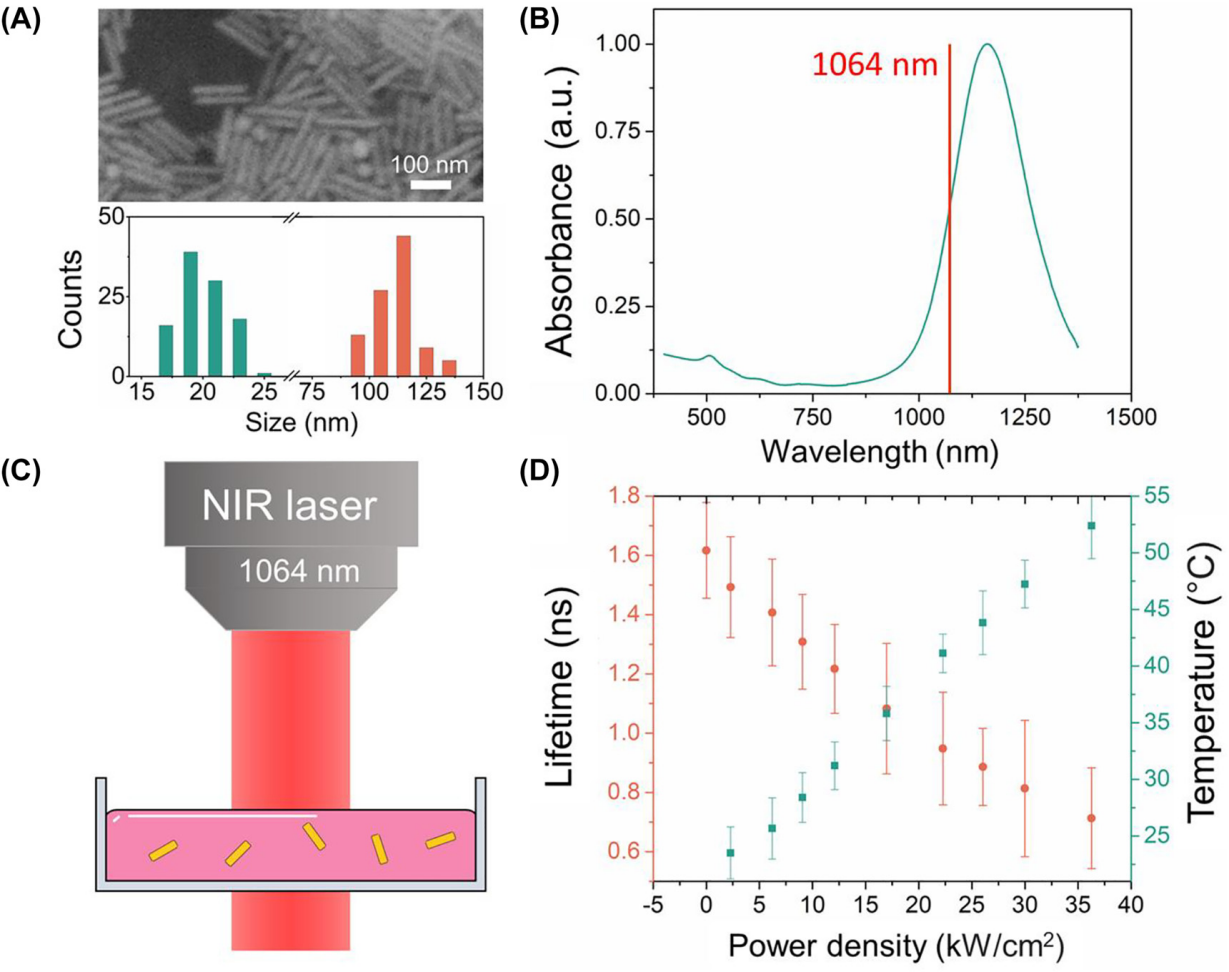
Au NRs characterization. (A) SEM images of AuNRs; (B) absorbance spectrum of Au NRs; (C) scheme of the experiment for fluorescence lifetime-based thermometry of RhB solution containing Au NRs; (D) the mean fluorescence lifetime of RhB solution in the presence of AuNRs (40 μg/mL) during NIR laser irradiation and the corresponding calculated temperature values.

The Au NRs are extensively used as nanoheaters for PTT due to their high biocompatibility and superior light absorbing properties [46]. Therefore, for the purpose of this study they were chosen as model nanoheaters. The morphological properties of the synthesized Au NRs were tailored to ensure that the peak absorbance lies in the NIR region (1160 nm) to cover the therapeutic window of biological tissues [47]. The surface of the obtained Au NRs was additionally modified with H_2_N-PEG-SH via ligand-exchange procedure to increase the solubility of these NPs in aqueous solutions and boost the cell association rate due to the positively charged surface [48]. The SEM images were obtained to evaluate the morphology of the synthesized Au NRs (Figure 2A). According to the SEM study, the length of the obtained Au NRs was 115.5 ± 14.8 nm, the width was 20.1 ± 1.9 nm, and thus their aspect ratio is ∼5.75. The synthesized Au NRs had two absorbance peaks: one at ∼510 nm, which corresponded to transversal plasmon, and the second at 1160 nm, which corresponded to longitudinal plasmon (Figure 2B).

Importantly, the presence of plasmonic NPs in the solution affects the fluorescence lifetime of RhB [49]. Therefore, the previously obtained calibration curve for a simple RhB solution cannot be used to determine the temperature of the solution containing the Au NRs. Consequently, in a manner similar to the previous experiment, a new dependence of fluorescence lifetime on temperature for a solution of RhB (100 μM) and Au NRs (20 μg/mL) was found:

(2) $$\tau =2.683\cdot {\text{e}}^{-0.0253\cdot T}$$

It can be seen that in the presence of Au NRs, the fluorescence lifetime has decreased significantly (Figure S4) due to non-radiative energy transfer between the RhB molecule and Au NRs. Additionally, it can be noticed that the increase of the solution temperature diminishes the difference in fluorescence lifetimes in the presence of Au NRs.

The obtained dependence then was used to evaluate the temperature change that occurs due to the NIR laser irradiation of Au NRs within RhB solution. For the following experiments a pulsed NIR laser with a wavelength of 1064 nm was used (Figure 2C). The fluorescence lifetimes were measured at various laser power densities (up to 36.3 kW/cm^2^). Several studies have shown that pulse laser in combination with Au NPs can cause cell death not only due to the increased temperature, but also due to explosion of the bubbles generated on the surface of plasmon nanoparticles. However, in our work the laser power densities are not high enough to induce this effect described elsewhere [50, 51]. Prior to each measurement, the solution was continuously irradiated for 60 s to stabilize the temperature. The light energy distribution within the laser spot follows Gaussian distribution; therefore, this was taken into account when determining laser power densities. Additionally, irradiated cells were precisely placed in the middle of the laser spot. The measured fluorescence lifetimes were recalculated into temperature values, using the previously obtained dependence (Eq. (2)) of RhB fluorescence lifetime on the temperature of the solution in the presence of Au NRs. According to the obtained data, the solution was gradually heated from the initial room temperature (23 °C) up to 52 °C (Figure 2D).

### 3.3 Intracellular temperature mapping

The fluorescence lifetime of the fluorophores depends on the properties of the surrounding media, which can be used to determine the intracellular temperature. However, the change in viscosity, pH and hydrophobicity of the surrounding media also affects the fluorescence lifetimes [43]. This means that the calibration curve for temperature estimation that we have derived previously for the water solution of RhB cannot be applied to measure and calculate intracellular temperature. The RhB mainly stains cell membranes, since it is highly soluble in them, and thus, the fluorescence lifetime increases compared to the fluorescence lifetime of RhB in water. Therefore, a new calibration curve for RhB-stained cells was necessary for further experiments.

The stained adherent B16-F10 cells were placed onto the heating plate, which was positioned on a piezo stage. Then the cell media was heated up to a certain temperature (from 32 °C to 46 °C), and the fluorescence lifetime measurements were performed. The detection rate was limited to 5% of the excitation repetition rate at the brightest pixel in order to avoid photon pile-up and prevent falsely shorter lifetimes. In each measurement, a single cell was scanned (step was equal to 1 μm) in order to accurately determine fluorescence lifetime distribution within a single cell at a certain temperature (Figure S7). Finally, the values were averaged and plotted against the temperature of the solution (Figure S8). It can be seen that the RhB that stained cells demonstrates longer fluorescence lifetimes compared to RhB water solution at the same temperatures. As it was mentioned previously, this effect can be explained by RhB exhibiting longer lifetimes in more viscous media, such as cell membranes [43].

Then the obtained data were fitted using the exponential fit, revealing the following relation between the fluorescence lifetime and intracellular temperature:

(3) $$T=61.02\cdot {\text{e}}^{-0.344\cdot \tau }$$

This equation was further used to determine the intracellular temperature in the latter experiments. It is important to underline that unlike the experiment with RhB water solution, the presence of internalized Au NRs inside of the cells did not affect the measured fluorescence lifetime values. Furthermore, this technique overall does not depend on the concentrations of the RhB used for cell staining. We have compared various concentrations of RhB (from 10 μM to 50 μM) and found no clear dependence of fluorescent lifetime values on the concentration of staining solution (Figure S9). Furthermore, since the dye was absent in the surrounding media and only cells were stained with RhB, fluorescence lifetimes and therefore intracellular temperatures were measured and not extracellular temperatures of the surrounding medium.

**Figure 3:**
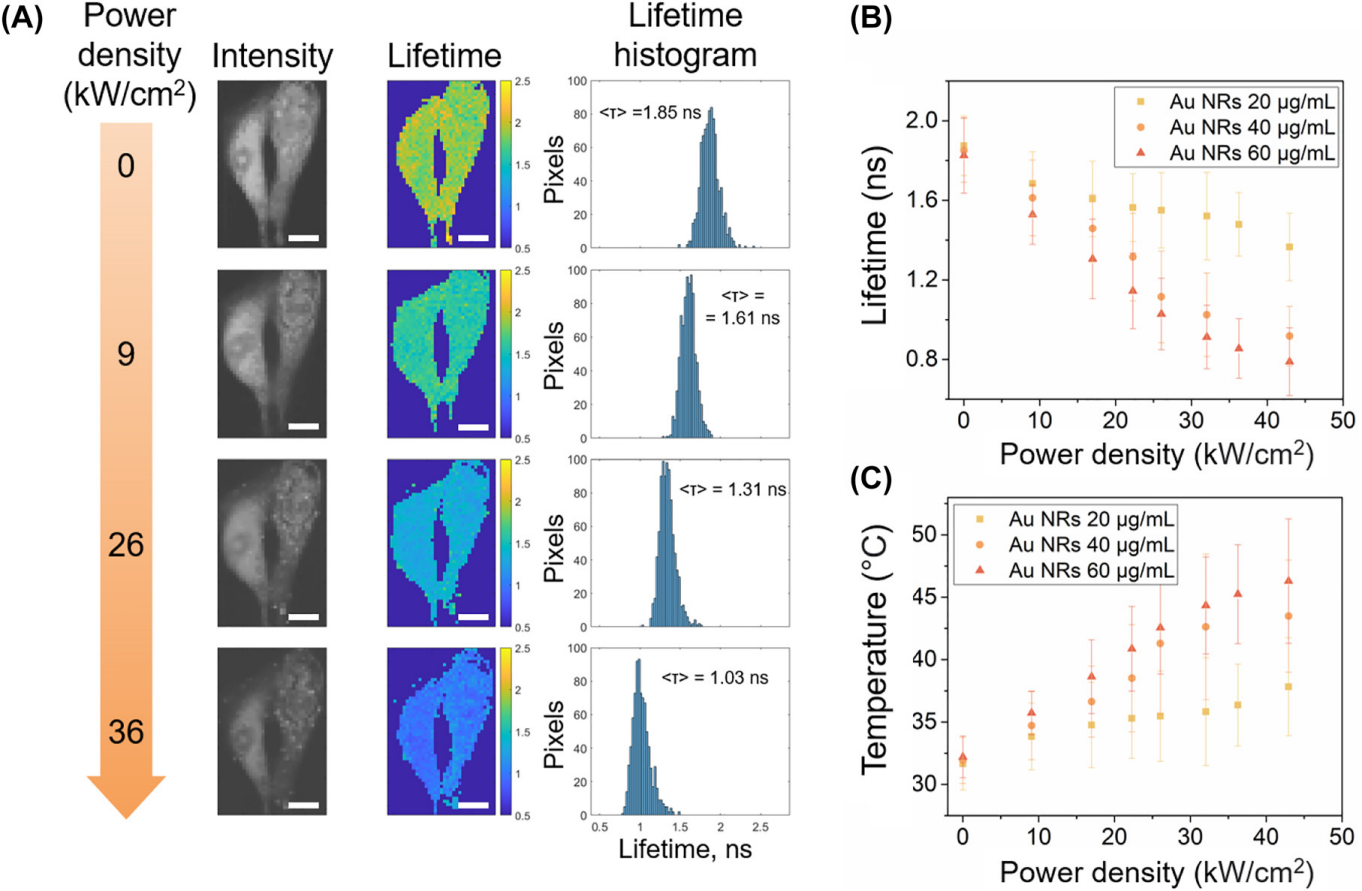
Fluorescence-based thermometry of B16-F10 cells. (A) Fluorescence lifetime images of B16-F10 cells incubated with AuNRs (40 μg/mL) and stained with RhB. Images were obtained while the cell was irradiated with an NIR laser with a specific power density. The image contains fluorescence intensity map, fluorescence lifetime map and the fluorescence lifetimes presented in histogram form. Scale bar corresponds to 5 µm; (B) average intracellular fluorescence lifetime of RhB depending on laser power density in the cells incubated with different concentrations of AuNRs (20, 40 and 60 μg/mL); (C) average intracellular temperatures depending on laser power density in the cells incubated with various concentrations of Au NRs. The data represent the mean and standard deviation of three independent samples.

To perform further experiments, it was necessary to choose appropriate concentrations of Au NRs. For this, the toxicity of Au NRs (from 1 μg/mL up to 60 μg/mL) was evaluated on murine melanoma cells B16-F10, and it was shown that none of these concentrations has cytotoxic effect (Figure S5). Thus, three concentrations were chosen for the further experiments: 20, 40 and 60 μg/mL. Furthermore, the cell uptake of Au NRs was evaluated using CLSM (Figure S6). It can be seen that some Au NRs are localized within the cells (presumably in lysosomes), forming aggregates. Another part of Au NRs is distributed on the surface of the cell membrane. Thus, it can be assumed that such a distribution may further lead to a uniform heating of a tumor cell. After 24 h of incubation, the Au NRs were added to the cells. On the following day, the cells were stained with 10 µM solution of RhB and placed on the heating plate in the fluorescent lifetime measuring setup. The temperature of the cell culture media was kept at 32 °C during the whole experiment. Prior to the fluorescence lifetime measurements of RhB-stained cells, they were irradiated with the NIR laser for 60 s to achieve uniform heating. After each successful scan, the power density was increased and a new fluorescence lifetime measurement was performed. Every fluorescence lifetime measurement yielded a fluorescence lifetime distribution within a single chosen cell for a specific laser power density (Figures 3A, S10, S11). The diameter of the laser beam (2 mm) was larger than the size of cells to ensure the even distribution of the laser power density. Afterwards, the values of the fluorescence lifetime within each cell were averaged and plotted against the laser power density. It can be seen that higher concentrations of Au NRs present in the cell culture medium lead to lower fluorescence lifetimes achieved during laser irradiation with the same power density and, therefore, higher intracellular temperatures (Figure 3B and C).

The measured average fluorescence lifetimes were converted into intracellular temperature values using the previously obtained calibration curve (Figure S8). It can be seen that the intracellular temperatures in the case of the lowest Au NRs concentration (20 μg/mL) do not exceed 36–37 °C even at the highest power densities, therefore, this concentration was deemed too low for further therapeutic use. Nonetheless, the intracellular temperatures during the irradiation with a power density equal to 42.9 kW/cm^2^ in the case of the highest Au NRs concentration (60 μg/mL) were as high as 46.3 ± 2.9 °C, which is enough to induce apoptosis in most of the treated cancer cells according to the published works [16]. Although the chosen method for temperature measurements may lack the accuracy compared to other works [32, 52], [53], [54], it demonstrates precision comparable to other fluorescence-based approaches. Furthermore, it can be used to estimate temperature distribution within the whole cell during the laser irradiation unlike nanoparticle-based measurements.

### 3.4 Apoptosis versus necrosis in 2D cell model

To evaluate the influence of the intracellular temperatures reached during laser irradiation of internalized Au NRs on the type of cell death, flow cytometry with Annexin V and 7AAD staining was performed. The goal of this experiment was to determine the intracellular temperatures sufficiently high to induce apoptosis in most of the cancer cells and to find the optimal concentrations of Au NRs, as well as corresponding laser power densities required to achieve them.

**Figure 4:**
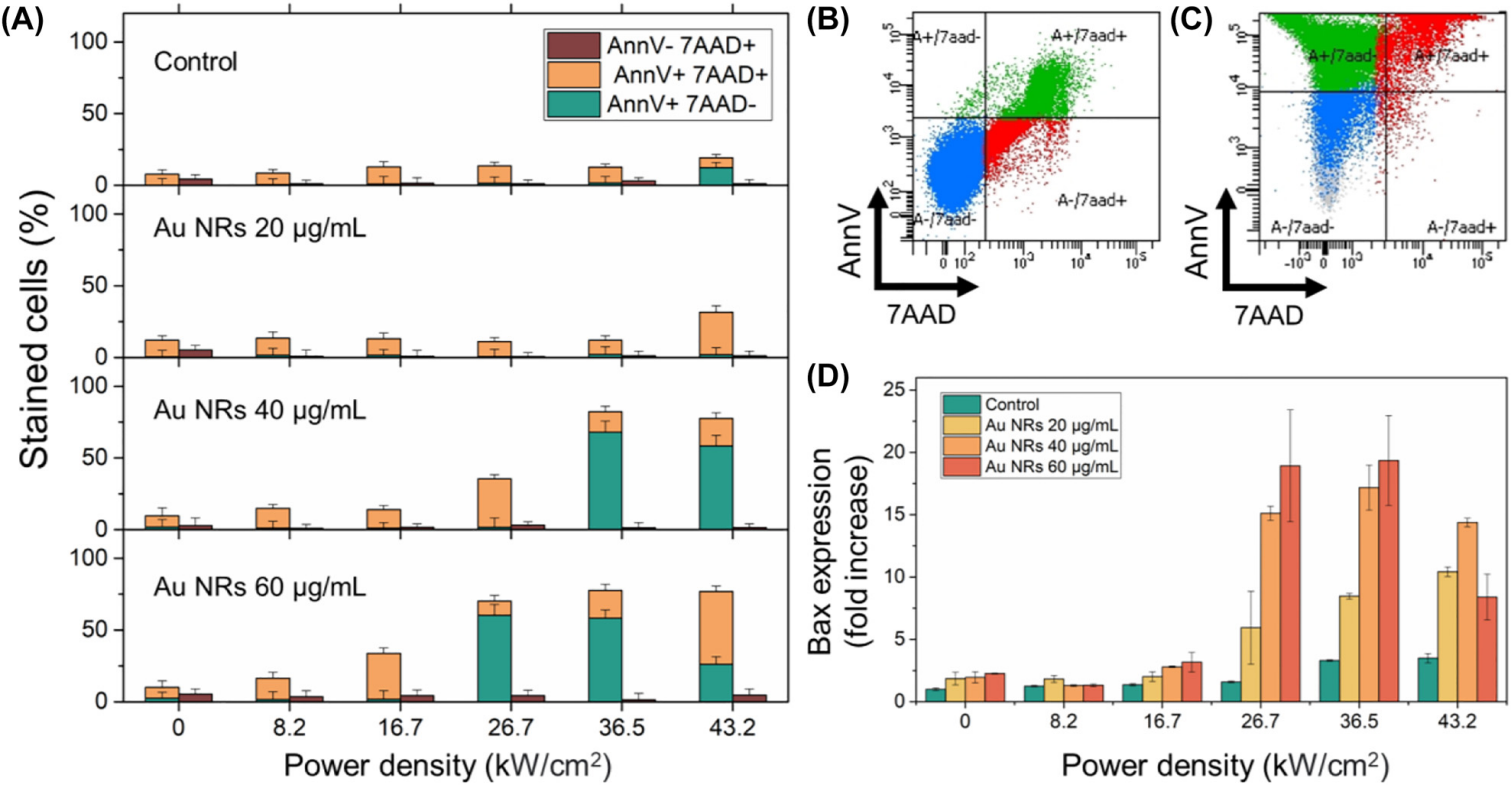
Viability of B16-F10 cells and Bax gene expression after laser irradiation. (A) The percentages of AnnV+, AnnV−, and 7-AAD+ cells incubated with different concentrations of Au NRs (20, 40, and 60 μg/mL) after irradiation with various laser power densities. (B, C) the flow cytometry data of Annexin V and 7AAD stained B16-F10 cells incubated with 40 μg/mL of Au NRs after no irradiation (B) and after irradiation with power density equal to 36.5 kW/cm^2^ (C). (D) Effect of Au NRs and laser irradiation on the expression level of Bax gene. In these experiments GAPDH was chosen as the housekeeping gene. The change in gene expression is shown as fold changes referring to untreated control cells. The data represent the mean and standard deviation of three independent samples.

For this, cells were incubated overnight with three different Au NRs concentrations (20, 40 and 60 μg/mL). Afterwards, each plate well with cells was irradiated with a certain laser power density (up to 43.2 kw/cm^2^) for 90 s. One hour after the irradiation, the cells were stained with Annexin V and 7-AAD and analyzed using flow cytometry. The plasma membrane of intact cells is composed of lipids that are asymmetrically distributed on the inner and outer leaflet of the membrane, with phosphatidylserine normally restricted to the inner leaflet and exposed during apoptosis [55]. Marking the phosphatidylserine with Annexin V allows distinguishing the apoptotic cells from the necrotic cells with permeable membranes that fail to exclude the 7-AAD Therefore, the results obtained from flow cytometry were interpreted in the following manner: cells negative in both channels (AnnV−/7-AAD−) were considered to be live, Annexin V positive and 7-AAD negative (AnnV+/7-AAD−) cells were considered to be in early apoptosis, cells positive in both channels (AnnV+/7-AAD+) – secondary necrotic (late apoptotic cells that suffer from the loss of membrane integrity in the absence of phagocytic cells) and, finally, Annexin V negative and 7-AAD positive (AnnV−/7-AAD+) cells were identified as necrotic (Figure 4B) [34].

From the obtained data, it can be seen that the amount of the apoptotic cells increases both with the increase of concentration of Au NRs added to the cells and the laser power density. At the power density equal to 43.2 kW/cm^2^ for both 40 and 60 μg/mL of Au NRs, more than 80% of all the cells were apoptotic. Additionally, it is interesting to notice that for 20 μg/mL of Au NRs; even the highest laser power density was not enough to induce significant cell death. However, if we take into account the data from the previous experiment, it becomes evident that for the case of 20 μg/mL of added Au NRs, the intracellular temperature did not exceed 36–37 °C, which explains why no apoptotic cells were detected. At the same time, at the highest laser power density (43.2 kW/cm^2^), the cells that were incubated with 40 and 60 μg/mL of Au NRs demonstrated intracellular temperatures equal to 43.1 ± 3.1 °C and 46.3 ± 2.9 °C, respectively. This temperature was sufficiently high to induce apoptosis in most of the cell population, with a varying number of cells undergoing secondary necrosis.

If we compare this data to the results of intracellular measurements, it becomes evident that significant cell apoptosis occurs once the temperatures reach more than 42 °C. As we increase the temperature further, the percentage of secondary necrotic cells increases up to 50% (60 μg/mL of Au NRs at 43.2 kW/cm^2^). Nonetheless, it is worth mentioning that the temperatures reached during PTT in all the samples were not high enough for primary necrosis to exceed more than 5.5% of the cells.

The cell apoptosis can be additionally determined by analyzing the expression level of the Bax gene in the cells using real time PCR. The Bax gene encodes BCL2L4 protein that induces cell death by altering cell mitochondria [56]. Various works have demonstrated the increase of expression level of Bax gene after heat-induced apoptosis [35–37]. In our case it can be seen that the expression of Bax gene was not increased in the cells that were treated with laser power densities below 26.7 kW/cm^2^ (Figure 4D). These results are in good agreement with the data from flow cytometry. As the intracellular temperature during laser irradiation begins to exceed 42 °C (40 μg/mL and 60 μg/mL of Au NRs at 26.7 kW/cm^2^) the Bax expression increases more than 15-fold (Figure 4D). This is reflected by the high percentage of apoptotic cells detected using flow cytometry in the samples treated with the same conditions. Interestingly, the Bax expression significantly decreases in the cells incubated with 60 μg/mL of Au NRs and treated with the power density equal to 43.2 kW/cm^2^. In this case the intracellular temperature exceeds 47 °C, which presumably leads to cell necrosis, thus, resulting in lower Bax gene expression levels.

### 3.5 Apoptosis versus necrosis in 3D cell model

In order to further investigate the influence of laser-induced Au NRs-mediated PTT, the following experiments were performed on a 3D spheroid tumor model of B16-F10 cells. Tumor spheroids are commonly used in drug development since they can replicate physiological cell environment to a higher extent compared to the usual 2D cell culture [41, 57, 58]. When it comes to PTT, the use of the spheroid tumor model can offer a new insight into the optimal laser power densities and Au NRs concentrations.

**Figure 5:**
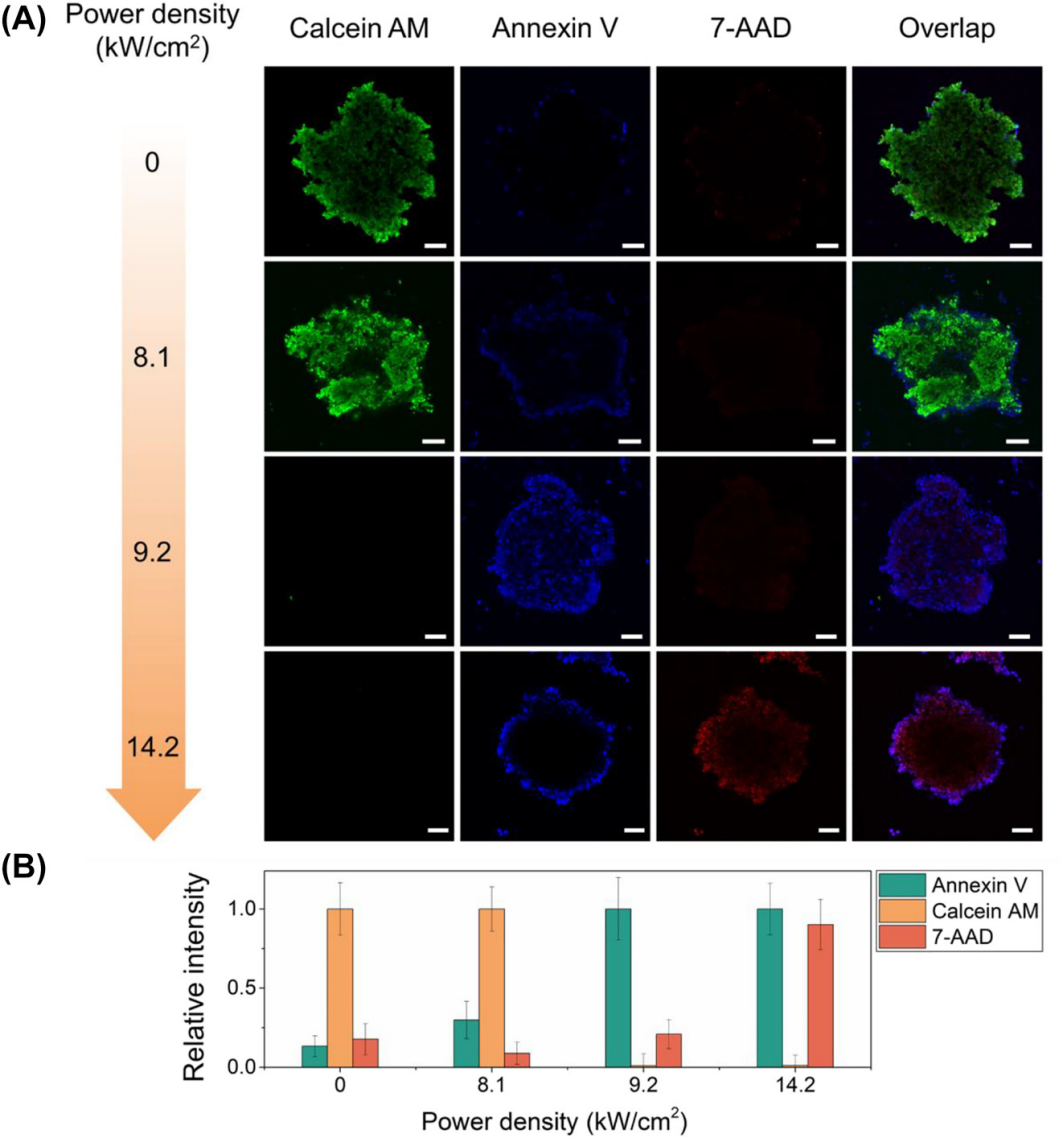
PTT of tumor spheroids. (A) The CLSM image of the tumor spheroid incubated with AuNRs (60 μg/ml) after PTT for various laser power densities. The live cells were stained with calcein AM (green), apoptotic cells – with APC Annexin V, and necrotic cells – with 7-AAD. Scale bar is equal to 5 µm. (B) Relative intensities of the corresponding fluorescence values calculated from the obtained CLSM images. The data represent the mean and standard deviation of three independent samples.

The tumor spheroids were incubated overnight with 60 μg/mL of Au NRs and afterwards subjected to PTT for 90 s, with laser power densities indicated above. Then, the tumor spheroids were stained with Calcein AM, APC Annexin V and 7-AAD. The live, apoptotic and necrotic cells were visualized using the CLSM (Figure 5A and B).

At laser power density equal to 8.1 kW/cm^2^, a certain amount of apoptotic cells on the periphery of the spheroid can be noticed with only a few 7-AAD+ cells. As power density was increased up to 9.2 kW/cm^2^, the number of Calcein AM positive cells dropped significantly, and the number of Annexin V positive cells greatly increased. Since the number of 7-AAD+ cells is insignificant, this means that almost all the cells died via apoptosis. Finally, at 14.2 kW/cm^2^, a large portion of spheroid cells became 7-AAD+ and, therefore, were considered to be necrotic. Overall, it can be seen that laser power densities required to induce a significant apoptosis in tumor spheroid were much lower compared to those required for 2D culture. This can be explained in the following manner: the tumor spheroid has decreased the heat exchange rate with the surrounding media compared to the 2D cell culture, and thus, during PTT, the same Au NRs concentrations and laser power densities may result in much higher intracellular temperatures. However, these temperatures could not be identified due to inherent limitations of the optical setup. The suggested fluorescence-based thermometry was developed to measure intracellular temperatures at nanoscale of 2D and 3D cell models, thus it cannot be utilized for temperature measurements of larger objects without significant alterations in the technique.

## 4 Conclusions

In this work, we have utilized a new approach for the evaluation of intracellular temperatures under the conditions similar to those of PTT. The developed thermometry method allowed us to evaluate the change of intracellular temperature due to laser-induced heating of internalized Au NRs. We have demonstrated that intracellular temperature above 42 °C was required to induce apoptosis in more than 80% of B16-F10 melanoma cells. At the same time, as intracellular temperature continues to rise, a greater number of cells undergo secondary necrosis. Overall, the developed thermometry approach allows precise estimation of the intracellular temperature during PTT, which, to the best of our knowledge, has not been reported elsewhere. These findings help to better understand how the temperature changes inside the cell during PTT and can be used in the future to further optimize the parameters for the development of novel PTT-based methods of cancer treatment.

## Supplementary Material

Supplementary Material Details
