# Supplementary material for: Fluorescence-based thermometry for precise estimation of nanoparticle laser-induced heating in cancerous cells at nanoscale
Source: Nanophotonics. 2022 Aug 15;11(18):4323–35. doi: 10.1515/nanoph-2022-0314 (PMC11501863; doi:10.1515/nanoph-2022-0314)
Supplement: Supplementary file 1 — Supplementary Material Details [file j_nanoph-2022-0314_suppl.docx]

***SUPPORTING INFORMATION***

**Fluorescence-based thermometry for precise estimation of nanoparticle laser-induced heating in cancerous cells at nanoscale**

Oleksii O. Peltek^1^, Eduard Ageev^1^, Pavel M. Talianov^1^, Anna D. Mikushina^2^, Olga S.Epifanovskaya^3^, Aliaksei Dubavik^4^, Vadim P. Veiko,^1^ Kirill Lepik^3^, Dmitry A. Zuev^1^, Alexander S. Timin^1,5,^*, Mikhail V. Zyuzin^1,^*

^1^School of Physics and Engineering, ITMO University, Lomonosova 9, 191002, St. Petersburg, Russian Federation

^2^Laboratory of Renewable Energy Sources, Alferov University, Khlopina 8/3, 194021, St. Petersburg, Russian Federation

^3^RM Gorbacheva Research Institute of Pediatric Oncology, Hematology and Transplantation, Pavlov University, Lva Tolstogo 6/8, 191144, St. Petersburg, Russian Federation

^4^Faculty of Photonics, Center of Optical Information Technologies, ITMO University, Birzhevaya liniya 4, 199034, St. Petersburg, Russian Federation

^5^Research School of Chemical and Biomedical Engineering, National Research Tomsk Polytechnic University, Lenin Avenue 30, Tomsk 634050, Russia

*correspondence to Dr. Mikhail V. Zyuzin and Dr. Alexander Timin at a_timin@mail.ru, timin@tpu.ru, mikhail.zyuzin@metalab.ifmo.ru

***Keywords***: laser-induced heating, necrosis/apoptosis, fluorescent nanothermometers, photodynamic therapy

**Table of contents**

[1. Materials 3](#_Toc109812378)

[2. Synthesis of functionalized Au NRs 3](#_Toc109812379)

[2.1. Synthesis of Au NRs 3](#_Toc109812380)

[2.2. Ligand exchange using H_2_N-PEG-SH 4](#_Toc109812381)

[2.3. UV-Vis spectrometry 4](#_Toc109812382)

[2.4. Au NRs concentration estimation 4](#_Toc109812383)

[2.5. AuNRs labeling with Cy5 4](#_Toc109812384)

[3. Experimental setup for fluorescence lifetime measurements 6](#_Toc109812385)

[4. External heating of Rhodamine B (RhB) solution 8](#_Toc109812386)

[5. Laser-induced heating of Au NRs in RhB solution 9](#_Toc109812387)

[6. Cells 10](#_Toc109812388)

[7. Toxicity studies 10](#_Toc109812389)

[8. Au NRs uptake 11](#_Toc109812390)

[8. External heating of cells 12](#_Toc109812391)

[9. Laser-induced heating of gold nanorods in cells 16](#_Toc109812392)

[10. Flow cytometry 18](#_Toc109812393)

[11. Bax gene expression analysis 20](#_Toc109812394)

[12. Formation of tumor spheroid 22](#_Toc109812395)

[13. Laser-induced heating of Au NRs in spheroid 22](#_Toc109812396)

[14. Statistical analysis 23](#_Toc109812397)

[References 23](#_Toc109812398)

# 1. Materials

*For Au NRs synthesis*: gold (III) chloride trihydrate (HAuCl_4_·3H_2_O, ≥99.9%, Sigma-Aldrich), ascorbic acid (AA, ≥99.0%, Sigma-Aldrich), sodium borohydride (NaBH_4_, 98%, Sigma-Aldrich), cetyltrimethylammonium bromide (CTAB, ≥99%, Sigma-Aldrich), sodium oleate (NaOL, > 97.0%, Sigma-Aldrich), amine-poly(ethylene glycol)-thiol (NH_2_-PEG-SH, MW 1.000, Laysan Bio, China) and Cy5-NHS ester (Luminoprobe) were all used without additional purification.

*For cell cultures*: Alpha Minimum Essential Medium (Alpha-MEM) was purchased from Biolot, Russia. Phosphate-buffered saline (PBS), and UltraGlutamine I were purchased from Lonza, Switzerland. Fetal bovine serum (FBS) was obtained from HyClone, USA. Trypsin-EDTA solution was purchased from Capricorn Scientific, Germany. Rhodamine B (RhB, ≥95%) and calcein acetoxymethyl (Calcein AM) were purchased from Sigma-Aldrich. AlamarBlue cell viability reagent was purchased from Invitrogen, USA. APC Annexin V apoptosis detection kit with 7-AAD was purchased from BioLegend, USA.

# 2. Synthesis of functionalized Au NRs

## 2.1. Synthesis of Au NRs

Au NRs were synthesized using seed-mediated growth approach and binary surfactant mixture according to the protocol developed by Xe et al. [1] The initial seed solution was prepared as follows: 5 mL of 0.2 M CTAB solution was added to 5 mL of 0.5 mM HAuCl_4_ under stirring. Afterwards, freshly prepared 0.6 mL of 0.01 M NaBH_4_ was diluted to 1 mL and then added to Au(III)-surfactant solution, which changed color to brownish. Stirring was stopped after 2 min and the formed seed solution was left undisturbed for 30 min prior further use. Meanwhile, the growth solution was prepared as follows. For this, 1.234 g of NaOL and 9 g of CTAB were added to 250 mL of water and then heated up to 50 °C until their complete dissolution. Afterwards, the solution was cooled down to 30 °C and 36 mL of 4 mM AgNO_3_ were added. The solution was gently mixed and left undisturbed for 15 min. Then 250 mL of 1 mM HAuCl_4_ solution was added and was left under stirring (700 rpm) for 90 minutes. Afterwards, 3 mL of HCl was injected to adjust pH and stirred for another 15 min. Finally, 1.25 mL of 0.064 M AA was added to the solution, quickly stirred for 30 s, and injected with 0.2 mL of previously prepared seed solution. The obtained solution was left for 12 h at 30 °C for Au NRs to grow. Following day, the solution was centrifuged at 7000 rpm for 30 min to remove the supernatant and resuspended in the same amount of deionized water

## 2.2. Ligand exchange using H_2_N-PEG-SH

Surface coating of CTAB-stabilized Au NRs was realized with ligand exchange procedure [2]. For this, 15 mL of the previously prepared Au NRs were centrifuged at 13000 rpm for 10 min. The supernatant was discarded and the Au NRs were resuspended in 0.5 mL of aqueous H2N-PEG-SH (10 mg/mL) using sonication for several minutes. Then Au NRs were centrifuged again, supernatant was removed, and another 0.5 mL of aqueous H_2_N-PEG-SH was added to Au NRs. The sonication was repeated, and the Au NRs were stored in it until future use.

## 2.3. UV-Vis spectrometry

The absorption spectra for the aqueous solutions of the functionalized Au NRs were measured in the 10 mm path quartz cuvettes using spectrophotometer Shimadzu UV-3600 (400-1300 nm interval) (**Figure 2B**).

## 2.4. Au NRs concentration estimation

The concentration of Au NRs was estimated using the Lambert–Beer law. For this, the absorption spectrum of the Au NRs was measured with a spectrophotometer Shimadzu UV-3600 (*l* =10 mm). The extinction coefficient for Au NRs was obtained from the literature: ε = 9.64*10^9^ M^-1^cm^-1^ [3]. The concentration was calculated in the following way:

$C=\frac{A}{\varepsilon l}=\frac{1.85}{9.64\cdot{10}^{9} M^{-1}{cm}^{-1}\cdot1cm}=0.192\cdot{10}^{-9}M$

## 2.5. Au NRs labeling with Cy5

To label the Au NRs modified with H_2_N-PEG-SH, 1 mg of Au NRs was redispersed in 900 μL of PBS buffer with pH=8.3-8.5. Afterwards 10 μg of Cy5-NHS ester dispersed in DMSO was added. Labeling was carried out at room temperature overnight. The following day Au NRs were washed thrice with water and finally redispersed in 1 mL of water.


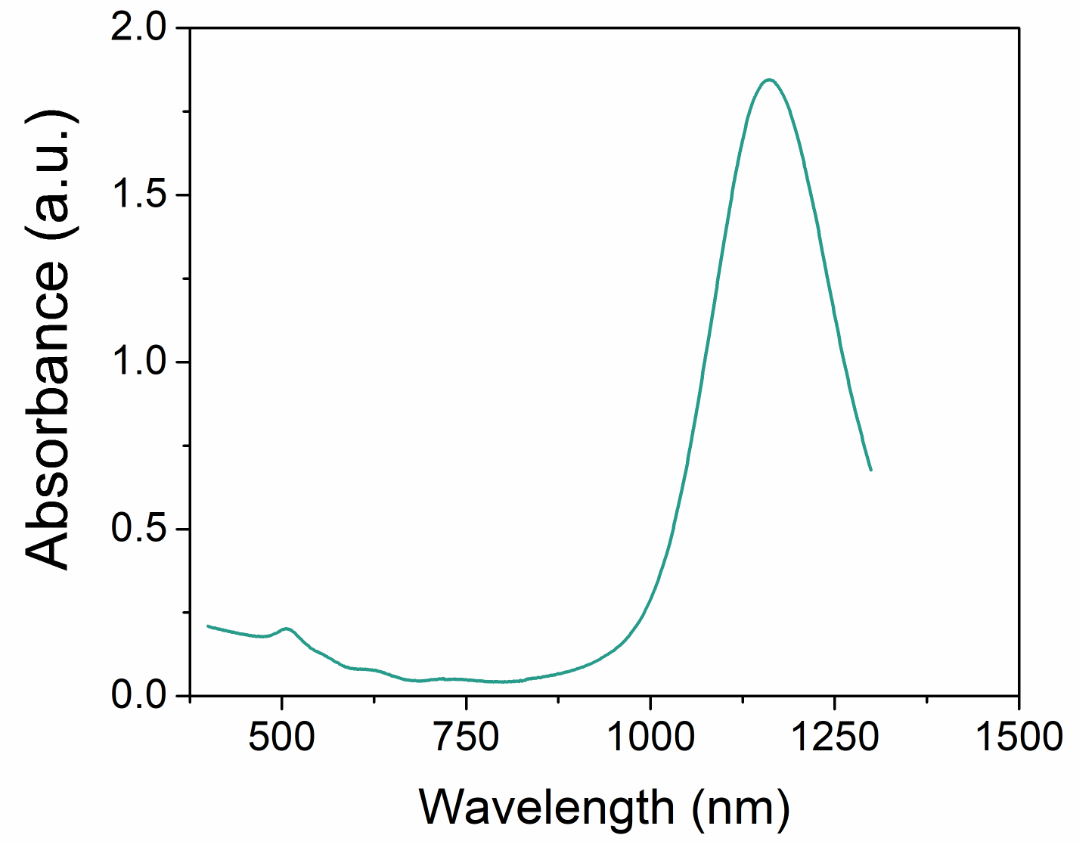


**Figure S1.** Absorption spectra of the Au NRs in water.

# 3. Experimental setup for fluorescence lifetime measurements


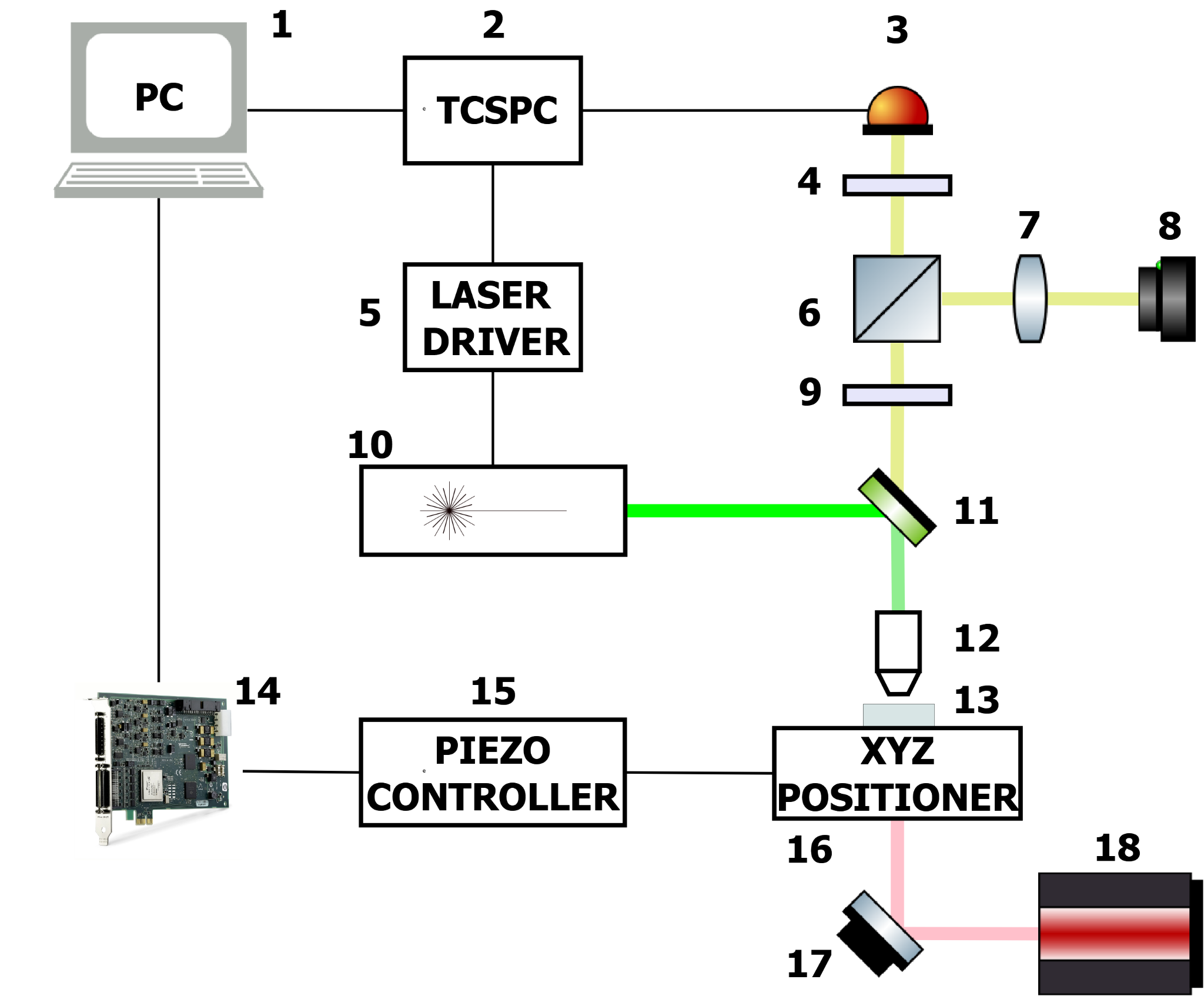


**Figure S2.** The experimental setup for FLIM measurements. 1 - PC, 2 - TCSPC module (Picoquant PicoHarp 300), 3 - SPAD (MPD PDM PD-100-CTC-FC), 4 - Longpass filter (Semrock BLP02-561R-25), 5 - Pulsed diode laser driver (Picoquant PDL 800-D), 6 - Beam splitter, 7 - Lens, 8 - Camera (Ximea xiQ), 9 - Notch filters (Semrock NF01-532U-25 & Thorlabs NF1064-44), 10 - Picosecond pulsed diode laser (Picoquant LDH-P-FA-530XL), 11 - Dichroic mirror (Semrock Di03-R532-t1-25x36), 12 - Objective (Olympus UPlanSApo 60X NA 1.20), 13 - Petri dish with sample, 14 - Multifunctional reconfigurable I/O device (NI PCIe-7841), 15 - Piezo controller (Piezosystem Jena NV 40/3 CLE), 16 - XYZ-positioner (Piezosystem Jena TRITOR 101 SGD), 17 - Mirror, 18 - Ytterbium fiber laser (IRE-POLUS ILMI-1-50).

To measure fluorescence lifetimes, time-correlated single photon counting (TCSPC) method was used. An experimental setup (**Figure S2**) is based on a home-built confocal microscope for fluorescence lifetime measurements with TCSPC. The light pulses of <100 ps FWHM from a 532 nm laser head (10) running at 1 MHz repetition rate are directed at the sample via appropriate optics. The fluorescence light is filtered against scattered excitation light using a dichroic mirror (11) and optical filters (4, 9). Then it is directed to the single-photon avalanche diode (SPAD) (3) via appropriate collection optics. A part of the light passes to the camera (8) through a removable beam splitter (6) and lens (7) for visualization purposes. The electrical signal obtained from the SPAD (3) is fed to the TCSPC electronics, Picoquant PicoHarp 300 (2), together with the electric sync signal from Picoquant PDL 800-D pulsed diode laser driver (5), which is used for the photon arrival time measurement. All measured time differences are sorted into a histogram for further analysis, aimed at the extraction of fluorescence lifetimes. To generate two-dimensional images, fluorescence lifetime imaging (FLIM) measurements are performed by raster scanning the sample with a TRITOR 101 SGD nanopositioner (16), driven by multifunctional reconfigurable I/O device (14) through NV 40/3 CLE piezo controller (15). The TCSPC electronics (2) use the time-tagged time-resolved (TTTR) data acquisition to synchronize with a scanning device for FLIM.

The spatial resolution is governed by the beam size of the green laser (λ = 532 nm) used for FLIM measurements at the focal point of objective (Olympus UPlanSApo 60X NA 1.20). According to the relation *d ≈ 1.22/NA*, the estimated full-width at half-maximum diameter of the beam focal spot size is 0.54 μm. Therefore, the spatial resolution is approximately 0.54 μm.

To provide heating of gold nanorods, an additional ytterbium laser source (18) is used. The light pulses of <140 ns FWHM from a 1064 nm fiber laser running at 100 kHz repetition rate are directed at the sample from the bottom via appropriate lenses and mirrors to collimate the beam and provide the beam diameter of 1 mm at the sample, while built-in electronics control laser power.

**
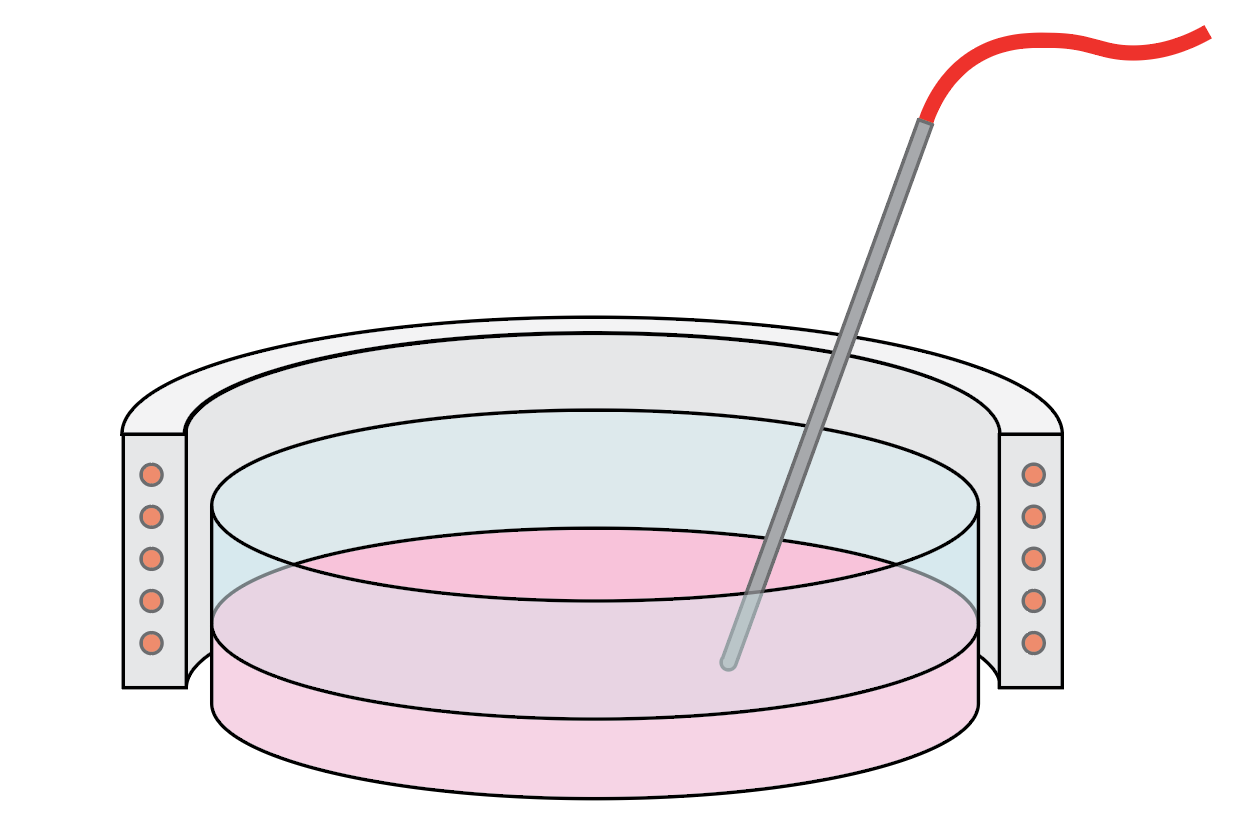
**

**Figure S3**. Temperature control unit

# 4. External heating of Rhodamine B (RhB) solution

In order to obtain a calibration curve describing the dependence of fluorescence lifetime of RhB on the temperature, 100 µM of aqueous RhB solution was used. Values of fluorescence lifetime were obtained using Picoquant Picoharp 300 time-correlated single photon counting system. Heating of RhB solution was realized using a custom-made temperature control system (**Figure S3**), in which the temperature of the solution was monitored via thermocouple. The values of fluorescence lifetimes of RhB were measured at various temperatures. The obtained data was fitted by single exponential fit using Matlab software (**Figure S4**):


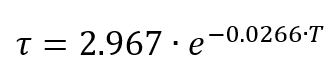


This fit was later used as a calibration curve to estimate RhB solution temperature based on the measured fluorescence lifetime values.


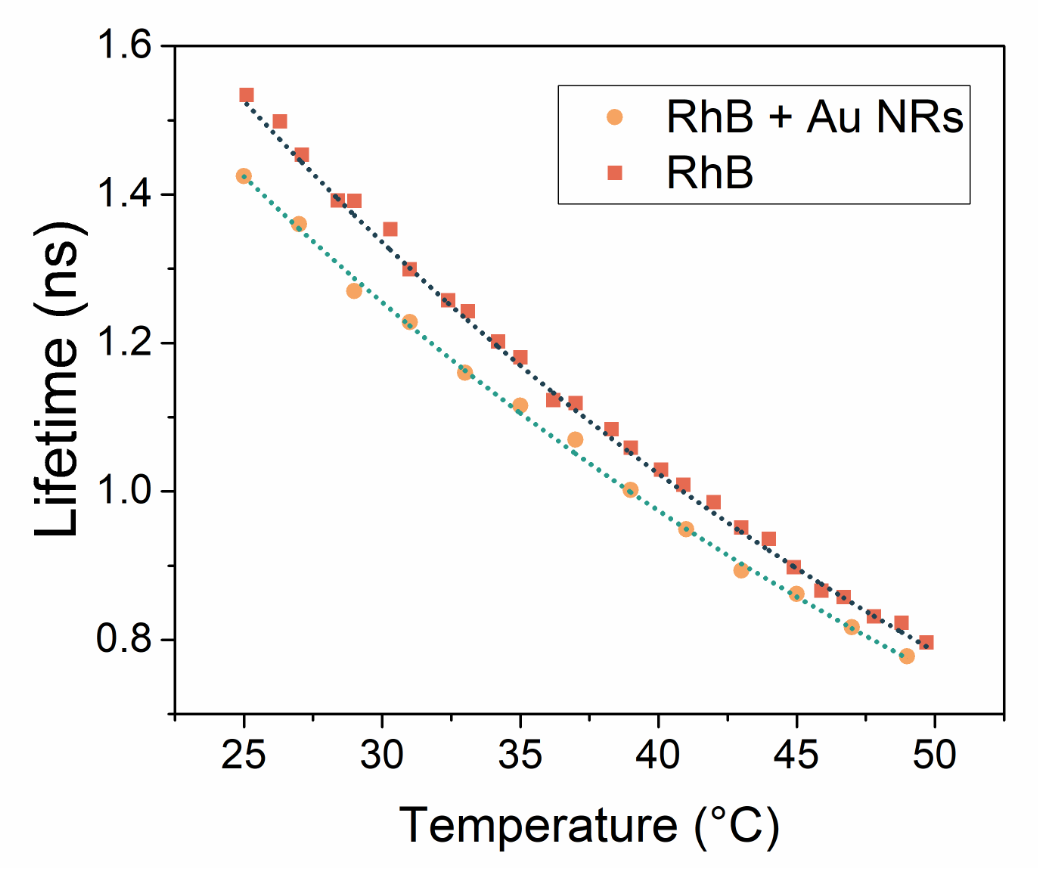


**Figure S4.** Temperature dependence of the mean fluorescence lifetime of RhB solution without (black dotted line) and in the presence of Au NRs (20 µg/mL).

# 5. Laser-induced heating of Au NRs in RhB solution

To evaluate the laser-induced heating of Au NRs, a freshly prepared 100 μM solution of RhB containing Au NRs with a final concentration of 20 µg/mL was used. Fluorescence lifetime measurements were carried out on the custom-made setup described in **3. Experimental setup for fluorescence lifetime measurements**. In order to evaluate the laser-induced heating of Au NRs in RhB solution, it was irradiated with a pulsed 1064 nm laser with different power densities (up to 36.3 kW/cm^2^). Before each measurement, the solution was continuously irradiated for 60 s to stabilize the temperature. In order to estimate the temperature of the solution during laser treatment, the previously obtained calibration curve for RhB solution containing Au NRs (20 µg/mL) was utilized (**Figure S4**):


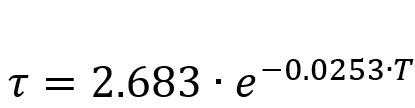


Using this dependence, the measured fluorescence lifetimes were recalculated into their corresponding temperature values.

# 6. Cells

Murine melanoma cell line (B16-F10 cells) was obtained from the American Type Culture Collection. Cells were cultured in AlphaMEM supplemented with 10% of vol. FBS and additional 2 mM UltraGlutamine I. The cell culture was maintained in a sterile humidified atmosphere containing 95% air and 5% CO_2_ at 37°C.

# 7. Toxicity studies

In order to evaluate the toxicity of Au NRs at different concentrations, AlamarBlue assay was performed. For this, B16-F10 cells were seeded into a 96-well plate at the amount of 1.0 x 10^3^ per well. The next day, Au NRs were added to each wellat different concentrations (1 µg/mL to 60 µg/mL). The final volume of cell medium in each well was 200 µL. After 24 h, cells were washed twice with PBS to remove the non-internalized Au NRs and the fresh medium supplemented with 10% vol. of AlamarBlue was added. The cell viability was evaluated according to the AlamarBlue Cell Viability protocol. Briefly, after 4 h of incubation the media containing AlamarBlue was analyzed by measuring absorbances of the media at 570 and 600 nm with UV−vis spectrophotometer (Thermo Scientific Multiskan GO). Experiment was performed three times to obtain mean value and the standard deviation. The mean value of cell viability was normalized to that of the cells that were not exposed to Au NRs.


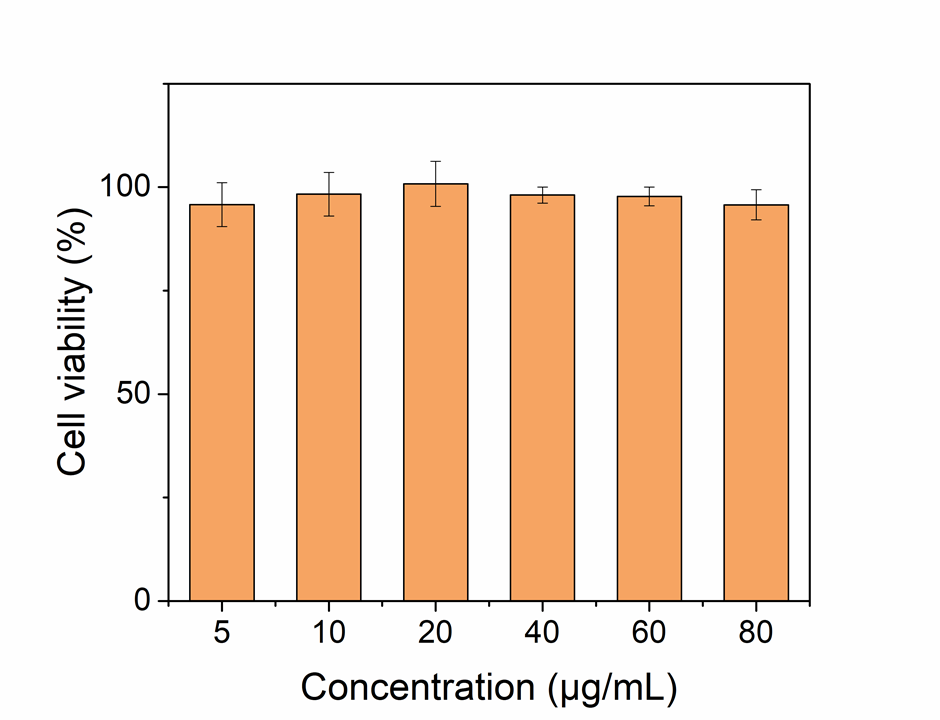


**Figure S5.** Viability histograms of B16 cells after the addition of Au NRs at different concentrations after 24h of incubation.

# 8. Au NRs uptake

To evaluate uptake and association of Au NRs with B16-F10 cells, cells were seeded into confocal cell imaging dishes (d = 35 mm, Eppendorf) at the amount of 1.0 x 10^4^ per dish. The next day, Au NRs labeled with Cy5 were added to the cells and cells were left overnight. On the following day, cells were stained with RhB. For this, 10 µL of 1 mM RhB solution was added to the old cell culture medium, so that the final concentration of RhB was equal to 10 µM. After 30 min, cells were washed twice with PBS to remove non-internalized Au NRs and excesses of RhB and left in PBS. Uptake was visualized using confocal laser scanning microscope (CLSM) Leica TCS SP8 (Germany). The confocal pinhole was set to 1 airy unit and images were taken with HC PL FLUOTAR 40x/0,80 PH2 Objective. Cells were scanned in 15 Z-planes with step 0.8 µm.


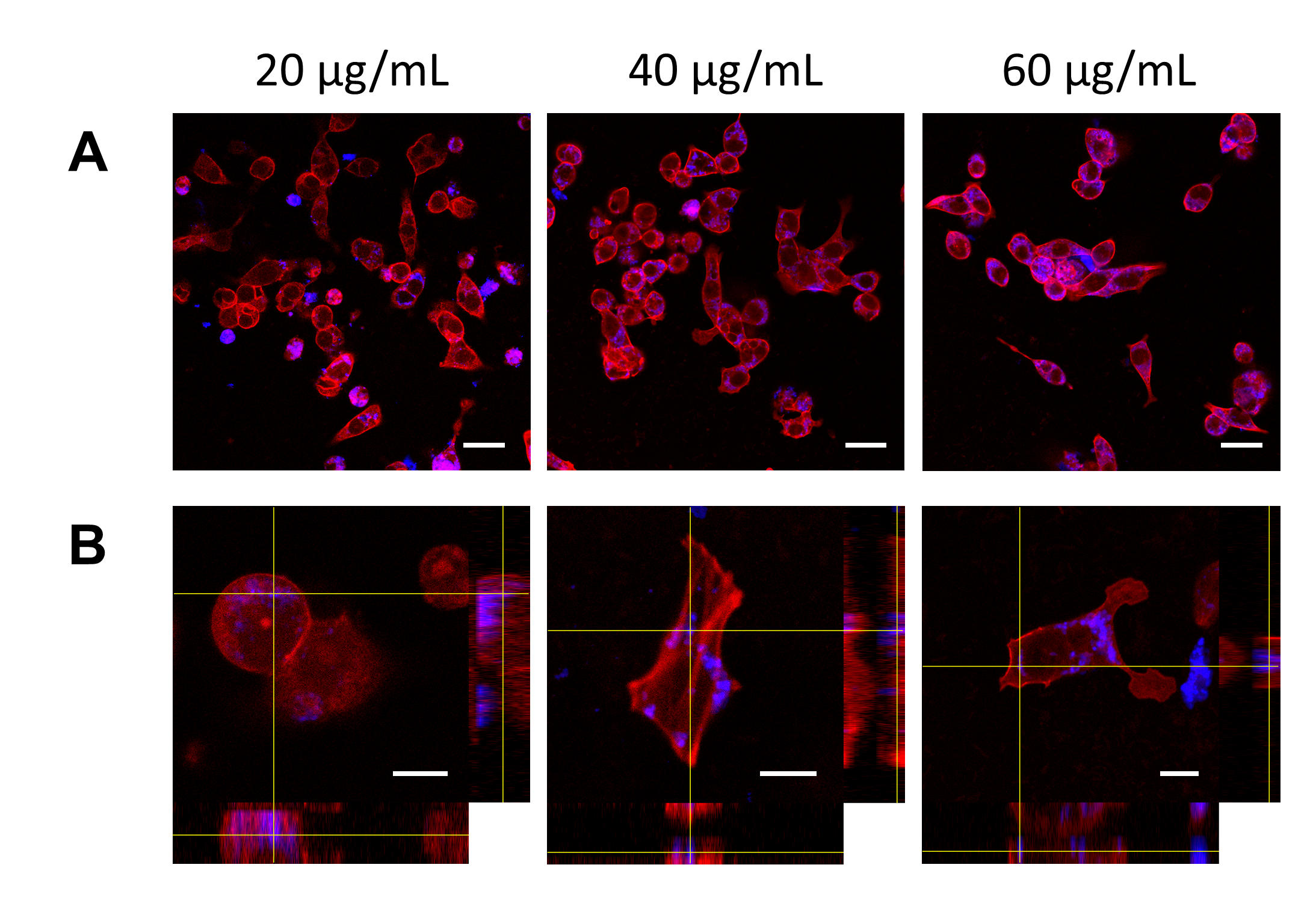


**Figure S6.** CLSM images of B16-F10 cells incubated with Au NRs. (A) Red fluorescence signal corresponds to cells stained with RhB (excited by a 512 nm laser) and blue fluorescence signal corresponds to Cy5 conjugated with Au NRs (excited by a 488 nm laser). Scale bar is equal to 25 µm. (B) Orthogonal view from different planes (x/y, x/z or y/z) of the CLSM images used to analyze the Au NRs uptake. Scale bar is equal to 10 µm.

# 8. External heating of cells

To estimate local cell temperatures, cells were previously stained with RhB. In order to obtain a calibration curve, showing the dependence of fluorescence lifetimes of RhB on the intracellular temperature, cells were seeded into confocal cell imaging dishes (d = 35 mm, Eppendorf) at the amount of 1.0 x 10^4^ per dish. The next day, Au NRs were added to the cells and cells were left overnight. On the following day, cells were stained with RhB. For this, 10 µL of 1 mM RhB solution was added to the old cell culture medium, so that the final concentration of RhB was equal to 10 µM. After 30 min, cells were washed twice with PBS to remove non-internalized Au NRs and left in PBS supplemented with 4% glucose. Fluorescence lifetime measurements were performed with custom made setup, which consisted of Picoquant PDL 800-D picosecond pulsed diode laser driver, Picoquant laser head LDH-FA 530XL, single photon avalanche diode (MPD PDM PD-100-CTC-FC) and Picoquant Picoharp 300 time-correlated single photon counting system. Before measurements, cells were heated up to a certain temperature (32 - 47 °C) by a custom-made temperature control system and kept for 5 min to ensure the uniformity of temperature distribution in the cell imaging dish. The obtained data was fitted by single exponential fit using Matlab software:


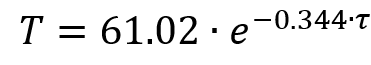


This fit was later used as a calibration curve to estimate mean intracellular temperature based on the measured fluorescence lifetime values (**Figure S7**).


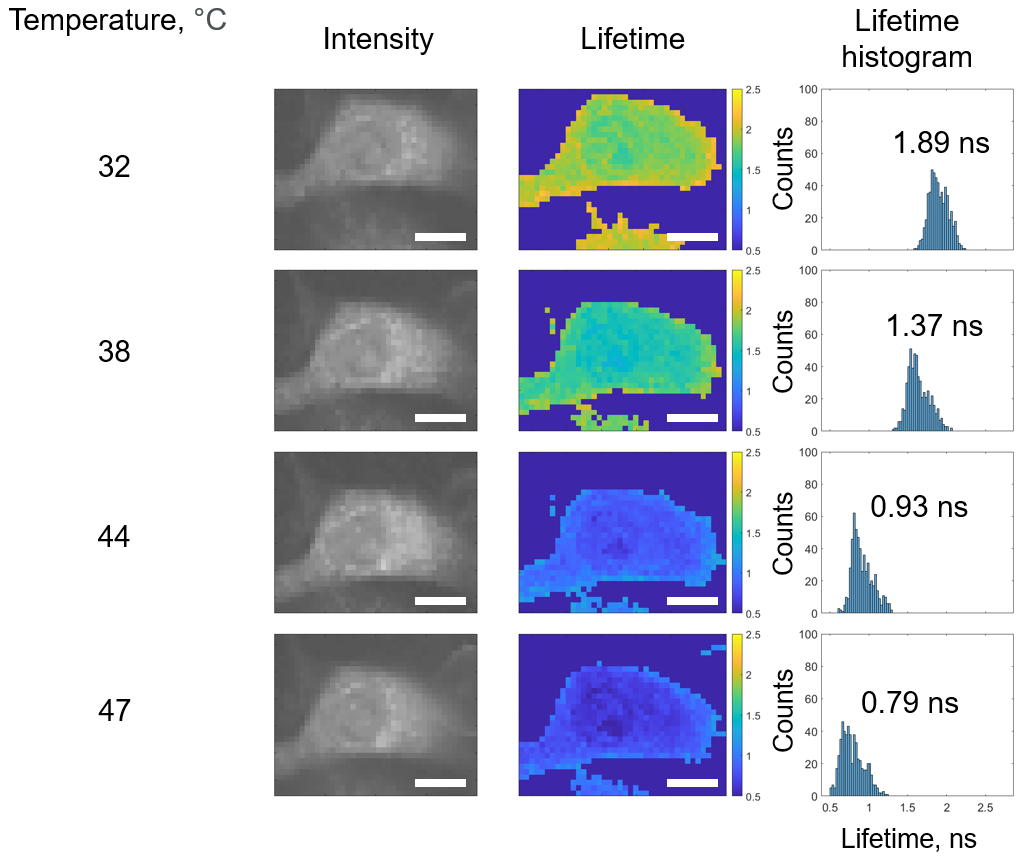


**Figure S7.** FLIM images of B16-F10 cells stained with RhB at the specific temperatures. The image contains fluorescence intensity map, fluorescence lifetime map and the lifetimes represented in histogram form. Scale bar is equal to 5 µm.


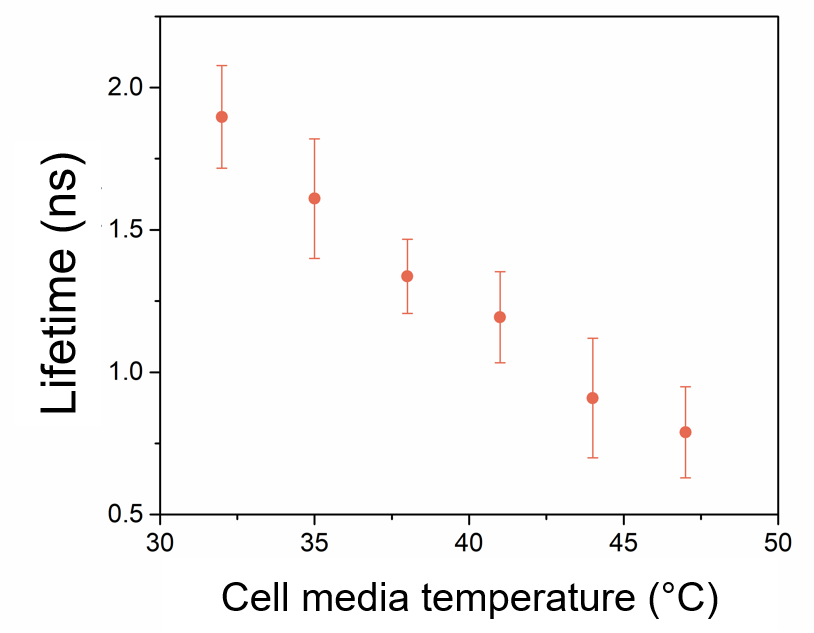


**Figure S8.** Temperature dependence of the mean fluorescence lifetime of RhB stained cells.


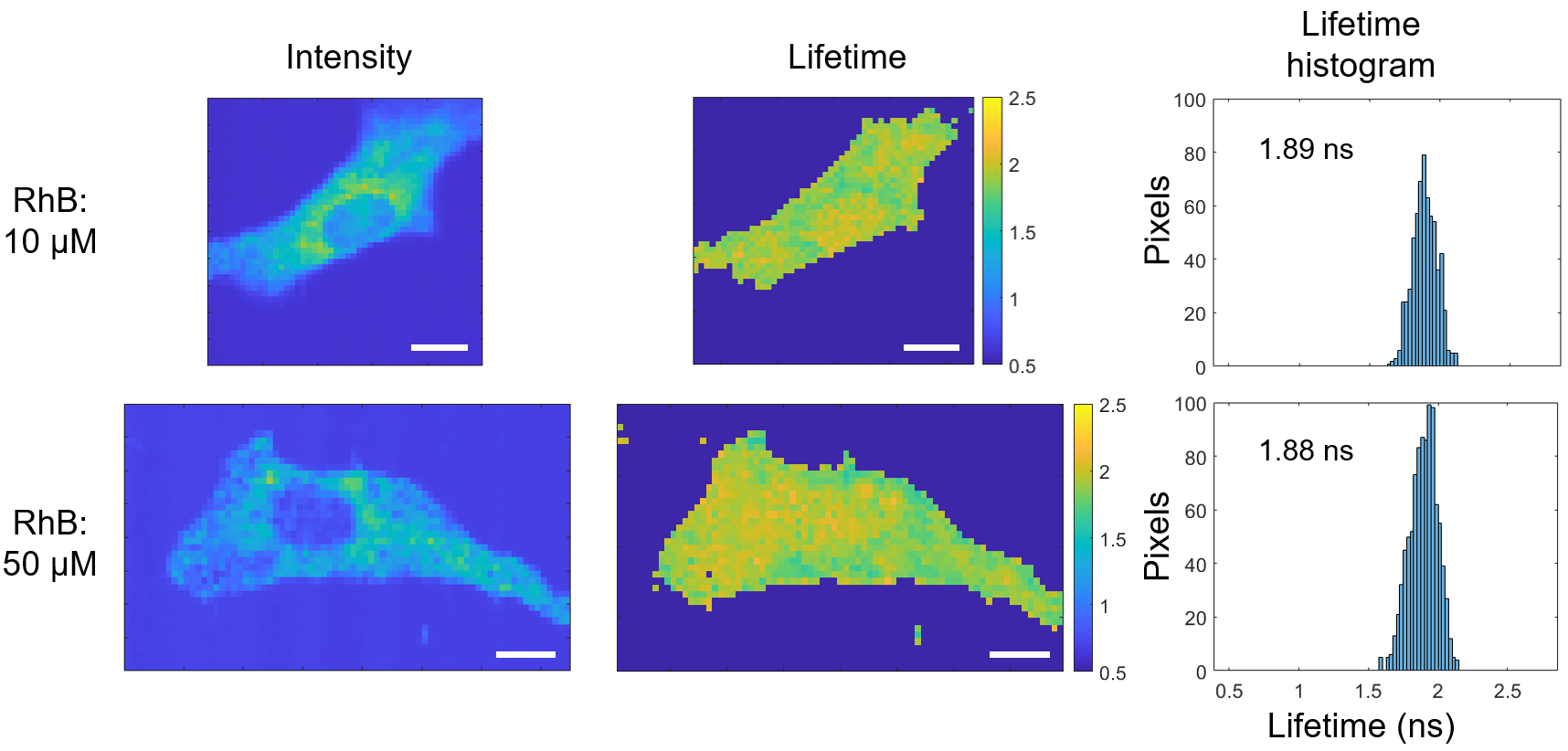


**Figure S9.** Different concentrations of RhB at the same external temperature (32°C). Scale bar = 5 um.

# 9. Laser-induced heating of gold nanorods in cells

In order to heat up cells with a NIR-laser, cells were seeded into confocal cell imaging dishes (d = 35 mm, Eppendorf) at the amount of 1.0 x 10^4^ per dish. The next day, Au NRs were added to the cells to obtain three different final concentrations of Au NRs in the culture media (20, 40 and 60 µg/mL). Cells were then incubated with Au NRs overnight. On the following day, cells were stained with RhB. For this, 10uL of 1 mM RhB solution was added to the old culture medium (final concentration was equal to 10 µM). After 30 min, cells were washed twice with PBS and left in PBS supplemented with 4% glucose. Fluorescence lifetime measurements were performed with the setup described in **Section 3**. During these measurements, the temperature of the cell medium was kept at 32 °C. Prior to measurement the cells were continuously irradiated for 60 s to stabilize the temperature. The previously found fit was used in these measurements as a calibration curve to estimate mean intracellular temperature based on the measured fluorescence lifetime values (**Figure S8**).


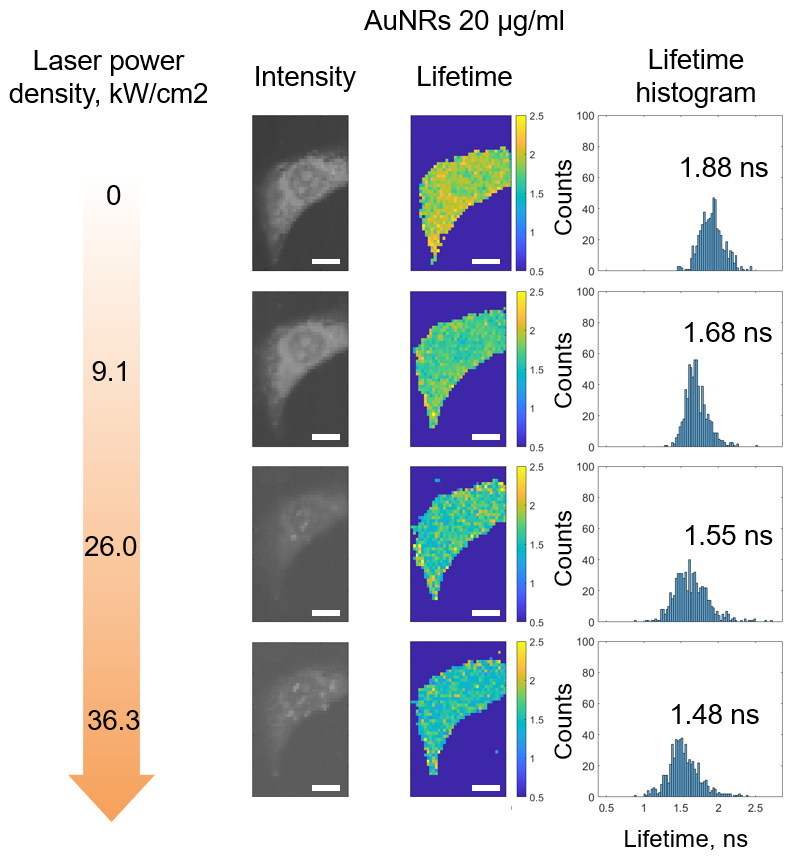


**Figure S10.** Fluorescence lifetime images of B16-F10 cells incubated with AuNRs 20 µg/ml and stained with RhB. Each image was obtained while the cell was irradiated with a NIR laser with a specific power density. The image contains fluorescence intensity map, fluorescence lifetime map and the fluorescence lifetimes represented in the form of a histogram. Scale bar is equal to 5 µm


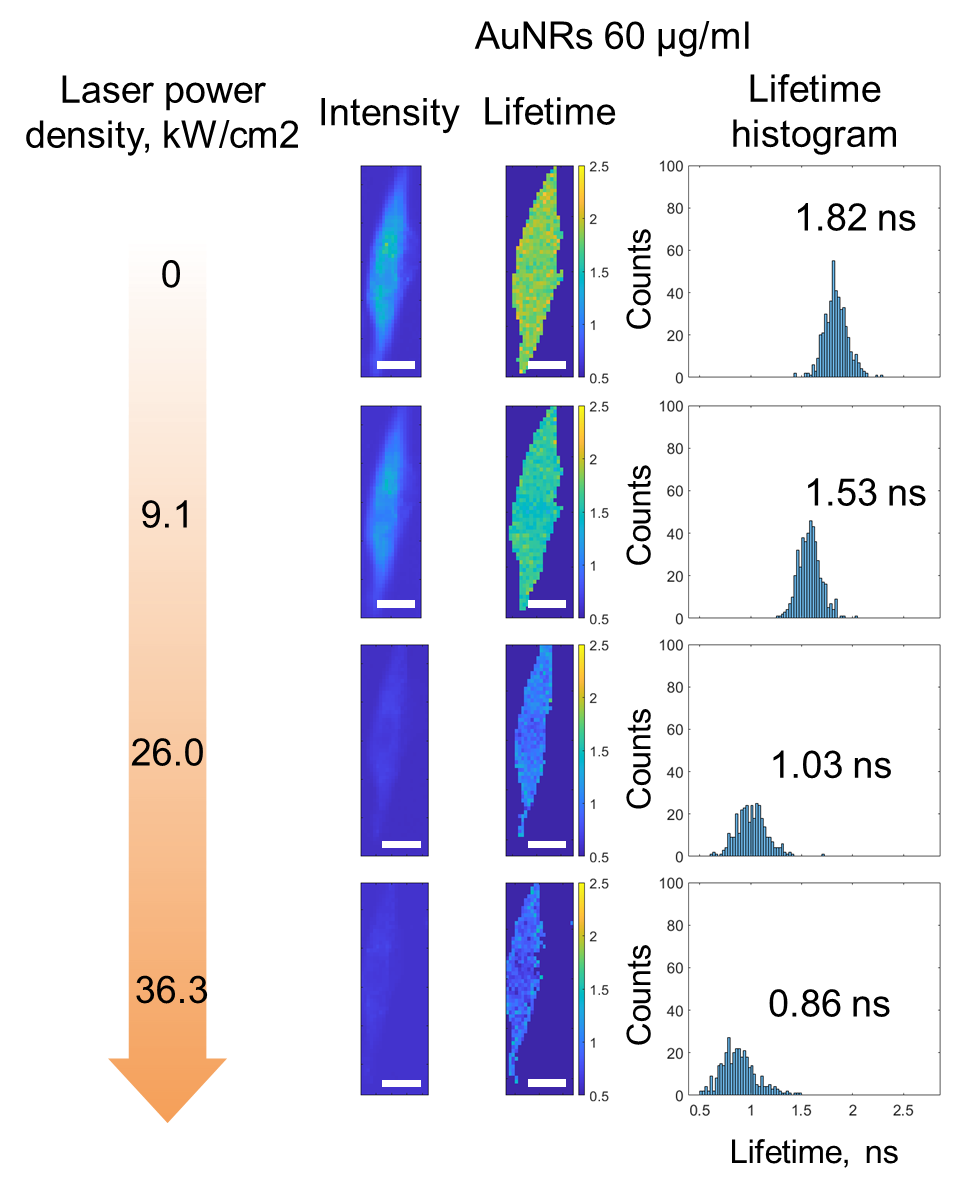


**Figure S11.** Fluorescence lifetime images of B16-F10 cells incubated with AuNRs 40 µg/ml and stained with RhB. Each image was obtained while the cell was irradiated with a NIR laser with a specific power density. The image contains fluorescence intensity map, fluorescence lifetime map and the fluorescence lifetimes represented in the form of a histogram. Scale bar is equal to 5 µm.

# 10. Flow cytometry

The apoptosis/necrosis assay was performed using flow cytometry (FACS Aria, BD, USA). For this, cells were seeded in 12-well plates at the amount of 1.0 x 10^5^ per well. Next day, Au NRs were added to each well at different concentrations (20 µg/mL to 60 µg/mL). The final volume of cell culture medium in each well was 1 mL. Afterwards, cells were washed twice with PBS and left in PBS supplemented with 4% glucose. Then each well was irradiated with a NIR-laser with different power densities (up to 43.2 kW/cm^2^) for 90 sec. One hour after irradiation, cells were detached using trypsin-EDTA (V = 200 µL per well) solution and stained with 7-AAD and APC Annexin V according to the protocol provided by the manufacturer (BioLegend, USA).


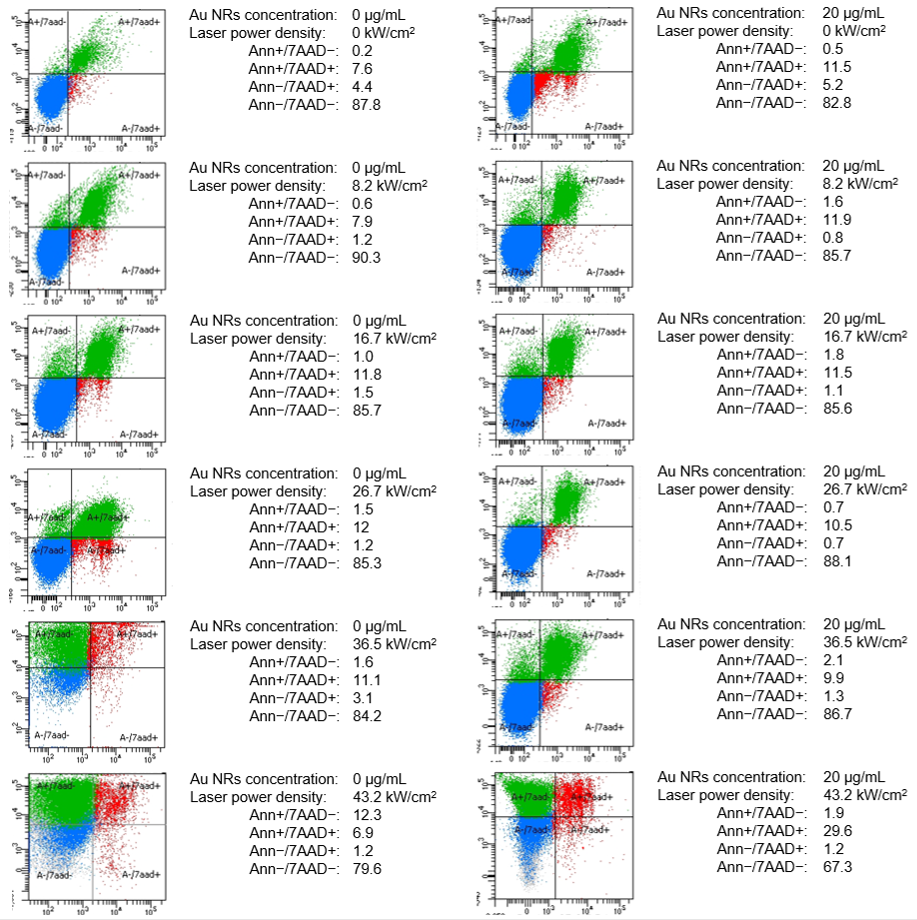


**Figure S12.** Apoptosis and necrosis analysis by flow cytometry using Annexin V/7AAD double staining. B16 cells incubated with different concentrations of Au NRs and then were irradiated with various laser power densities.


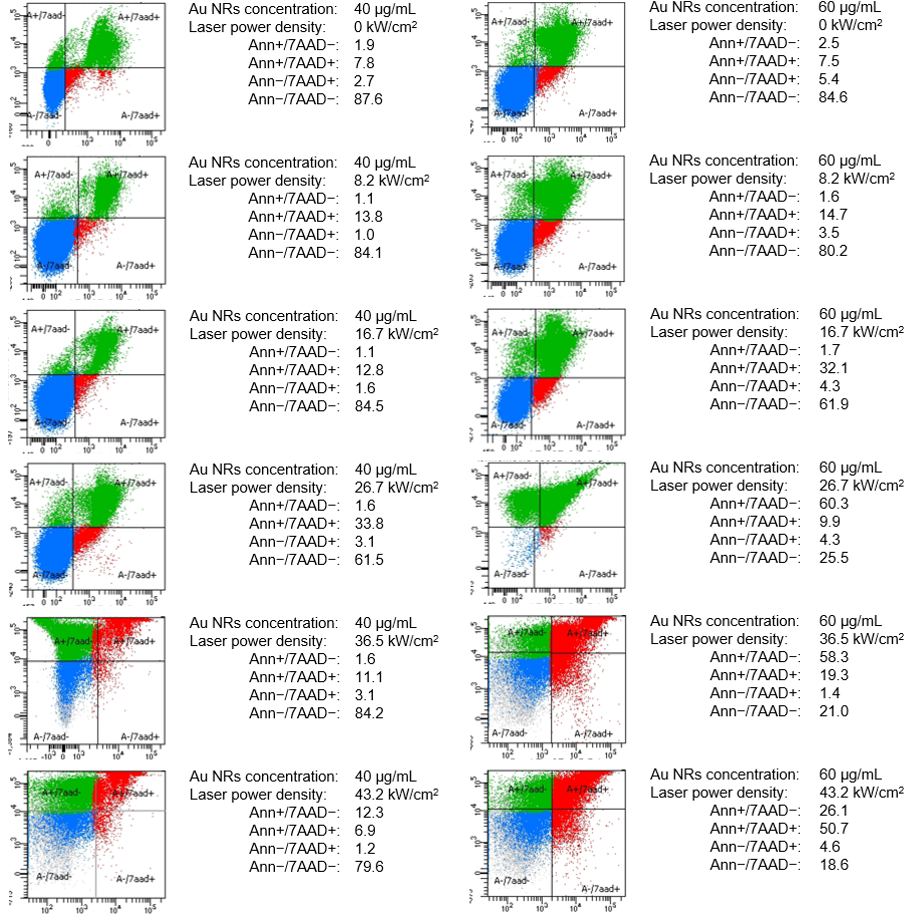


**Figure S13.** Apoptosis and necrosis analysis by flow cytometry using Annexin V/7AAD double staining. B16 cells incubated with different concentrations of Au NRs and then were irradiated with various laser power densities.

# 11. Bax gene expression analysis

*RNA extraction*

Total RNA was extracted from 1 x 10^6^ B16-F10 cells treated in vitro at different points of examination, using an RNA extraction kit (Evrogen, Russia) according to the manufacturer’s instructions. Briefly, the cells were homogenized by solution ExtractRNA containing phenol and guanidinium isothiocyanate (GITC). After 15 min incubation and 10 min centrifugation at 15 000xg to the supernatants was added chloroform and the solution was centrifuged for 15 minutes at 4°C. RNA from the aqueous phase was precipitated in 100% isopropanol. After centrifugation isopropanol was removed and 75% ethanol was added to the residual pellet. After additional centrifugation ethanol was removed and the RNA residue was left to dry for several minutes at room temperature. After drying the samples were diluted in RNAse free water.

*Synthesis of cDNA and Quantitative real time (qRT-PCR)*

cDNA was synthesized from the total RNA using MMLV RT kit (Evrogen, Russia). The reaction was performed with oligo(dT)_15_-primer and M-MLV reverse transcriptase. Synthesized cDNA was used as a template for amplification of Bax gene using real-time PCR. Gene GAPDH (Glyceraldehyde 3-phosphate dehydrogenase) was used as an endogenous housekeeping control. All experiments were carried out in triplicate. The primers used for amplification are shown in **Table 1**. Each gene was amplified in a separate 20 mL reaction using Taq-polymerase and SYBR Green I (Evrogen, Russia) The reaction was performed using thermal cycler Eco Real-Time PCR System (Eco™ Software v5; Illumina, Inc., San Diego, CA, USA). The following thermal profile was used: PCR initial activation step at 95 °C for 3 min; 40 cycles of a three-step cycling consisting of the denaturation at 95 °C for 15 s, the annealing at 54 °C for 20 s and the extension at 72 °C for 20 s. The results of these genes were normalized to GAPDH. RNA levels (relative fold change) were determined using the method described by Livak et al. [4]

**Table 1**. Primer sequences used for real-time PCR.

| Gene | Sense 5’ -> 3’ | Antisense 5’ -> 3’ |
| --- | --- | --- |
| GAPDH | CAAGGTCATCCATGACAACTTTG | GTCCACCACCCTGTTGCTGTAG |
| Bax | CACGTCCACGATCAGTCACG | GCTTCTTGGTGGACGCATCC |

# 12. Formation of tumor spheroid

Spheroids were obtained using the “hanging drop” technique described by Timmins et al. [5] Briefly, 15 μL of culture medium which contained 5000 B16-F10 cells were carefully dropped onto the inside cover of a 35 mm Petri dish. The dish itself was filled with 1.5 mL PBS to prevent evaporation of cell media. The cover of the dish was placed back onto the Petri dish, and cells were incubated at 37 °C and 5 % CO_2_. The cell culture medium inside the drop was changed each 3 days and after 6 days the spheroid formation was complete.

# 13. Laser-induced heating of Au NRs in spheroid

In order to laser-heat 3D tumor spheroids, Au NRs were added to the previously prepared spheroids at a final concentration of 60 µg/mL. Next day, spheroids were washed twice with PBS to remove the non-internalized Au NRs and carefully transferred to a separate Petri dish prior to the irradiation. Then, each spheroid was irradiated for 90 sec with a NIR-laser with different power densities (up to 14.2 kW/cm^2^). After irradiation, each spheroid was washed with 20 µL of BioLegend's cell staining buffer and transferred to the Annexin V Binding Buffer, which contained 0.2 µM calcein AM, 5 µL APC Annexin V (per 100 µL of Buffer) and 5 µL 7-AAD (per 100 µL of Buffer). Then cells were incubated for 15 min at room temperature before the visualization with a CLSM (Carl Zeiss LSM 710). In order to visualize cells stained with calcein AM (live cells), argon laser emitting at 488 nm was used. To visualize cell nuclei stained with 7-AAD (dead cells), helium-neon laser emitting at 633 nm was used. The cells stained with APC Annexin V (apoptotic cells) were visualized with helium-neon laser emitting at 633 nm. The confocal pinhole was set to 1 airy unit and images were taken with an Objective EC Plan-Neofluar 40x/1.30 Oil DIC. Spheroids were scanned in 10 Z-planes with step 2 µm.

# 14. Statistical analysis

The accuracy of temperature measurement was estimated by standard deviation using the following equation:


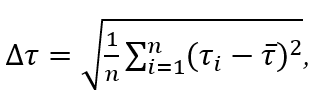


where n is the number of lifetime measurements for the same external temperature, τ_i_ is the experimental value of the fluorescence lifetime and τ is the mean value of all fluorescence lifetime measurements for the same power.

Afterwards, the obtained standard deviation for fluorescence lifetimes was converted into the temperature values using the previously found calibration curves.

# References

[1] X. Ye, C. Zheng, J. Chen, Y. Gao, C.B. Murray, Using Binary Surfactant Mixtures To Simultaneously Improve the Dimensional Tunability and Monodispersity in the Seeded Growth of Gold Nanorods, Nano Lett. 13 (2013) 765–771.

[2] A.R. Muslimov, A.S. Timin, V.R. Bichaykina, O.O. Peltek, T.E. Karpov, A. Dubavik, A. Nominé, J. Ghanbaja, G.B. Sukhorukov, M.V. Zyuzin, Biomimetic drug delivery platforms based on mesenchymal stem cells impregnated with light-responsive submicron sized carriers, Biomater. Sci. 8 (2020) 1137–1147.

[3] C.J. Orendorff, C.J. Murphy, Quantitation of Metal Content in the Silver-Assisted Growth of Gold Nanorods, J. Phys. Chem. B. 110 (2006) 3990–3994.

[4] K.J. Livak, T.D. Schmittgen, Analysis of Relative Gene Expression Data Using Real-Time Quantitative PCR and the 2−ΔΔCT Method, Methods. 25 (2001) 402–408.

[5] N.E. Timmins, L.K. Nielsen, Generation of Multicellular Tumor Spheroids by the Hanging-Drop Method, in: H. Hauser, M. Fussenegger (Eds.), Tissue Engineering, Humana Press, Totowa, NJ, 2007: pp. 141–151.
